# Systematic review of research barriers, facilitators, and stakeholders in long-term care and geriatric settings, and a conceptual mapping framework to build research capacity

**DOI:** 10.1186/s12877-023-04318-x

**Published:** 2023-10-04

**Authors:** Quoc Dinh Nguyen, Marie-France Forget, Xi Sophie Zhang, Catherine Richer, Renata Podbielski, Pierrette Gaudreau, Philippe Desmarais

**Affiliations:** 1 Division of Geriatrics, Centre hospitalier de l’Université de Montréal, Montreal, Canada; 2 Centre de recherche du Centre hospitalier de l’Université de Montréal, Montreal, Canada; 3 Department of Medicine, Université de Montréal, Montreal, Canada; 4 Department of General Medicine, Centre intégré de santé et de services sociaux du Centre- Sud-de-l’île-de-Montréal, Montreal, Canada; 5 Department of Family and Emergency Medicine, Université de Montréal, Montreal, Canada; 6 Direction of Teaching and the CHUM Académie, Centre hospitalier de l’Université de Montréal, Montreal, Canada

**Keywords:** Long-term care, Nursing home, Geriatric acute care, Barriers and facilitators, Research methods, Underrepresentation

## Abstract

**Background:**

Older adults are underrepresented in research. Heterogeneity of research processes in this population, specifically in long-term care (LTC) and geriatric acute care (GAC), is not well described and may impede the design, planning, and conduct of research. In this study, we identified, quantified, and mapped stakeholders, research stages, and transversal themes of research processes, to develop a mapping framework to improve research capacity by better characterizing this heterogeneity.

**Methods:**

Multicomponent mixed methods study. An environmental scan was used to initiate a preliminary framework. We conducted a systematic literature search on processes, barriers, and methods for clinical research in GAC and LTC to extract and update stakeholders, research stages, and themes. Importance and interactions of elements were synthesized via heatmaps by number of articles, mentions, and content intersections.

**Results:**

For our initial framework and environmental scan, we surveyed 24 stakeholders. Of 9277 records, 68 articles were included in our systematic review and allowed us to identify 12 stakeholders, 13 research stages, 17 transversal themes (either barriers, facilitators, general themes, or recommendations), and 1868 intersections. Differences in relative importance between LTC and GAC emerged for stakeholders (staff, managers vs. caregivers, ethics committees), and for research stages (funding, facility recruitment vs. ethics, individual recruitment). Crucial themes according to specific stakeholders were collaboration for the research team; communication, trust, and human resources for managers; heterogeneity for patients and residents. A heatmap framework synthesizing vital stakeholders and themes per research stage was generated.

**Conclusions:**

We identified and quantified the interactions between stakeholders, stages, and themes to characterize heterogeneity in LTC and GAC research. Our framework may serve as a blueprint to co-construct and improve each stage of the research process.

**Supplementary Information:**

The online version contains supplementary material available at 10.1186/s12877-023-04318-x.

## Background

Older adults remain underrepresented in clinical research, even as their share of the population is growing rapidly [1–3]. As epitomized during the COVID-19 pandemic, vulnerable older adults are disproportionately affected by adverse health outcomes but comprise only a small proportion of participants in clinical trials [4, 5]. Beyond the physical dimension, older adults’ vulnerability may also pertain to the psychological, relational, moral, sociocultural and existential domains [6]. Longstanding research underrepresentation occurs across disciplines and throughout this continuum of vulnerability in older adults [7–10]. As older adults are heterogenous [11, 12], underrepresentation has been more prevalent for those at the far end of vulnerability and frailty due to restricted eligibility criteria based on cognitive and functional impairment, comorbidities, logistical issues, or life expectancy [13–15].

Long-term care (LTC) residents and patients receiving care in geriatric acute care units (GAC) face multiple barriers in both the recruitment phase and the general conduct of research [16–19]. In a systematic review on research challenges in LTC facilities, Lam et al. reported eight main themes related to characteristics of facility/owner/administrator, resident, staff, family caregiver, investigator, ethical or legal concerns, methodology and budgetary considerations, and proposed numerous solutions within these domains [17]. Furthermore, Bowling et al. developed a framework, the *5Ts*, to support the inclusion of older adults in research: Tools, Target Population, Team, Time, and Tips to improve research communication [20]. Advocacy by researchers in aging has also led to recommendations and changes at the policy and regulatory levels [21, 22]. Unfortunately, as the COVID-19 pandemic has demonstrated, there remains a critical need to improve the scope and magnitude of research conducted in care settings specific to older adults [23].

Whereas heterogeneity in older adults and its impact on research is well described [24–26], the heterogeneity of research questions, context and settings, stakeholders, governance, training, and readiness of research in LTC and GAC may have been underappreciated [27]. Aging is a multifaceted phenomenon where the interactions and intersections of people, place, time, and content give rise to diverse manifestations [28]. It may not be possible to provide a list of barriers, facilitators, or recommendations that would be applicable to all research on older adults [29], in LTC [17, 19, 30] or in GAC [18]. The wide variation of contexts where this research occurs remains an under-investigated area which may have hindered the capacity to conduct research successfully. We believe that applying a multidimensional and systems lens to research in aging may empower researchers, residents, patients, and other stakeholders. Recognizing the complexity, the need for flexibility, and the opportunities for collaboration, may promote alignment between researchers and others stakeholders to increase and improve research capacity in this population [31].

In this multicomponent study, we thus carried out an environmental scan, a systematic mixed methods review and synthesis to develop a conceptual mapping framework that identifies and quantifies the critical stakeholders, stages, and themes for research in LTC and GAC. The framework aims to serve as a blueprint for researchers and stakeholders to enhance facilitators and reduce barriers when designing and co-constructing specific studies in these settings.

## Methods

We used a hybrid mixed methods approach comprising three steps to reach our final conceptual framework: an environmental scan and initial framework, a best fit qualitative systematic review, and a quantitative synthesis [32–34].

### Environmental scan and initial framework

The first step was an environmental scan with stakeholder representatives [35]. We conducted brief semi-structured telephone interviews with participants of diverse clinical and research backgrounds with regards to expertise and experiences to identify their perspectives on relevant (i) stakeholders, (ii) research stages, and (iii) themes, facilitators and barriers when conducting research in geriatric acute care or mixed (GAC) settings or in LTC [36]. Participants at this stage were recruited by purposive and snowball sampling and included patient-partnership representatives, researchers and staff in aging and long-term care, clinicians (neurology, geriatrics, family medicine, nursing, pharmacy), and managers. Based on these interviews and content expertise from our team of researchers (comprising female and male researchers with complementary credentials, experiences, and knowledge in geriatric medicine, family medicine, fundamental and clinical research, occupational therapy, and mixed methods), an initial conceptual framework focusing on the interrelations and intersections between elements was proposed and agreed upon by all investigators (Supplementary Fig. 1).

### Search strategy, study selection, extraction of themes and framework update

In the second step, we conducted a systematic search of the literature to identify articles pertaining to our research question in 6 databases (including Medline, EMBASE, CINAHL, PsycINFO, EBM Reviews) and 11 grey literature websites. The search was conducted on September 27, 2021; the strategy was designed by a librarian (RP) and was limited to adults. We included contemporary articles published in English or French between 2000 and 2021 pertaining to the processes, methods, collaboration, or networks (i.e., collective initiatives or groups with the purpose of facilitating or conducting research, both ad hoc and formally structured) for clinical research in aging in middle- or high-income settings. Exclusion criteria were studies not related to GAC or LTC units, articles not pertaining to research issues, narrative accounts on research, and articles on research priorities or themes. After removing duplicates, two reviewers (PD, QDN) screened titles and abstracts independently and in duplicate. The Supplementary Methods present the detailed search strategy and queries. Since this component of the study was a qualitative systematic review, it was not registered on PROSPERO.

Year of publication, study jurisdiction, methodology, and study population (i.e., LTC or GAC setting) of selected articles were extracted. Articles were read in full and analyzed to systematically update the initial listing of relevant stakeholders, research stages, and themes (PD, MFF, QDN). We coded the content in the results and discussion sections of articles based on our conceptual framework and definitions [32]. The coded content was mapped to stakeholders, research stages, transversal themes, and, when relevant, whether the content was a facilitator or a barrier to conducting research. An initial pilot phase was conducted for five articles and two validation meetings were held. Themes that emerged during the coding phase were incorporated to iteratively update the framework.

### Quantitative synthesis and final conceptual framework and maps

The third and final step was to describe and quantify the interrelations between stakeholders, stages, and themes. We used individual articles as the primary unit of analysis to quantify (i) the number of articles describing specific stakeholders, research stages, and transversal themes; whether these were perceived as barriers or facilitators, (ii) the common mention (dual intersection) of specific stakeholders and research stages in units of coded content, and (iii) the triple intersection of specific stakeholders, research stages, and themes. Each intersection was only counted at most once per article. We initially stratified results according to GAC and LTC units; when results were not qualitatively different, they were combined for concision. Our final conceptual framework map represents the interactions with two- and three-dimension heatmaps featuring stakeholders, stages, and themes. Analyses were conducted using R 4.2.0 (R Foundation for Statistical Computing, Vienna). The study was approved by the IRB of the *Centre de recherche du Centre hospitalier de l’Université de Montréal*.

## Results

### Environmental scan and initial framework

We conducted interviews with 24 stakeholders in GAC and LTC: 7 staff members, 6 clinical researchers, 6 researchers, 2 managers, 1 caregiver stakeholder representative, 1 research staff member, and 1 research network representative. Content analysis produced a preliminary list of stakeholders and research stages (Supplementary Table 1), and transversal themes (Table 1). These elements were structured to produce the initial conceptual framework and interaction map with stakeholders and research stages as axes and transversal themes at their intersection (Supplementary Fig. 1).

**Table 1:** Research stakeholders, stages, and transversal themes

Research stakeholders	Transversal themes	Defining and related concepts
Research team Residents and patients Caregivers Staff Facilities and centers Ethics review committees Managers Management and regional authorities Research networks and groups Funding agencies and institutes Foundations Trainees	*Alignment	Fit between project objectives and the perception and needs of stakeholders
	Appropriation	Project ownership, mobilization, buy-in, local leadership and championing
	Collaboration	Interdisciplinary work, team,
	Communication and trust	Relationship, positive perception
	Ethics	Ethical issues and considerations, capacity, consent (including proxy), autonomy, power of attorney, privacy, confidentiality
	Fragmentation	Isolation, centralization, network, coordination
	*Funding	Grants, securing funds, costs for research
**Research stages**	*Governance	Central authority, administrative and operations rules, corporate responsibility
Study planning and protocol Study design and methods Funding Ethics approval Facility or center recruitment Individual participant recruitment Consent Intervention Data collection and outcomes Analyses Knowledge transfer Sustainability Training		
	Heterogeneity	Variability in population and centers, personalization, adaptability, adjustments, feasibility and logistics with specific population
	Human resources	Staff shortage, staff turnover
	Inefficiency	Duplication, redundancy, delays, friction, and obstacles
	Information technologies	Implementation and availability of technology, digital data infrastructure
	*Legal and regulations	Legislation, statutes, legal protection
	Material resources	Physical space, equipment
	*Reputation	Public perception, threats to reputation
	Standardization	Harmonization, consensus, guidelines, best practices, simplification
	Training	Knowledge, lack of knowledge, uncertainty

**Notes.** *These themes were identified in conducting the systematic review

### Systematic literature search and article characteristics

The database search identified 9277 records; 8 were identified through other sources. Following duplicate removal, 7014 records were screened, and 132 articles were assessed in full for eligibility. We included 68 articles which are presented in Supplementary Table 2; Supplementary Fig. 2 presents the PRISMA flow chart. Thirty (45%) articles were published by authors from the USA and 23 (34%) from the United Kingdom. Approximately a third of articles were reviews (n = 23, 34%) or qualitative (n = 21, 32%); 51 (75%) concerned LTC and 27 (25%) had GAC or mixed populations. Supplementary Table 3 presents the full characteristics of articles.

### Stakeholders, research stages, transversal themes

Content coding from the 68 articles yielded 12 stakeholders, 13 research stages, and 17 transversal themes (content coded as barriers, facilitators, general themes, or recommendations). Compared to our initial framework, 1 new stakeholder emerged (i.e., facilities and centers); researchers and research staff, as well as professional caregiving staff and non-professional caregiving staff, were respectively collapsed into a single group. Data collection and outcomes were combined as single stage, and 5 new transversal themes emerged: alignment, funding, governance, legal and regulations, and reputation. Table 1 presents the themes and their defining and related concepts.

**Fig. 1:**
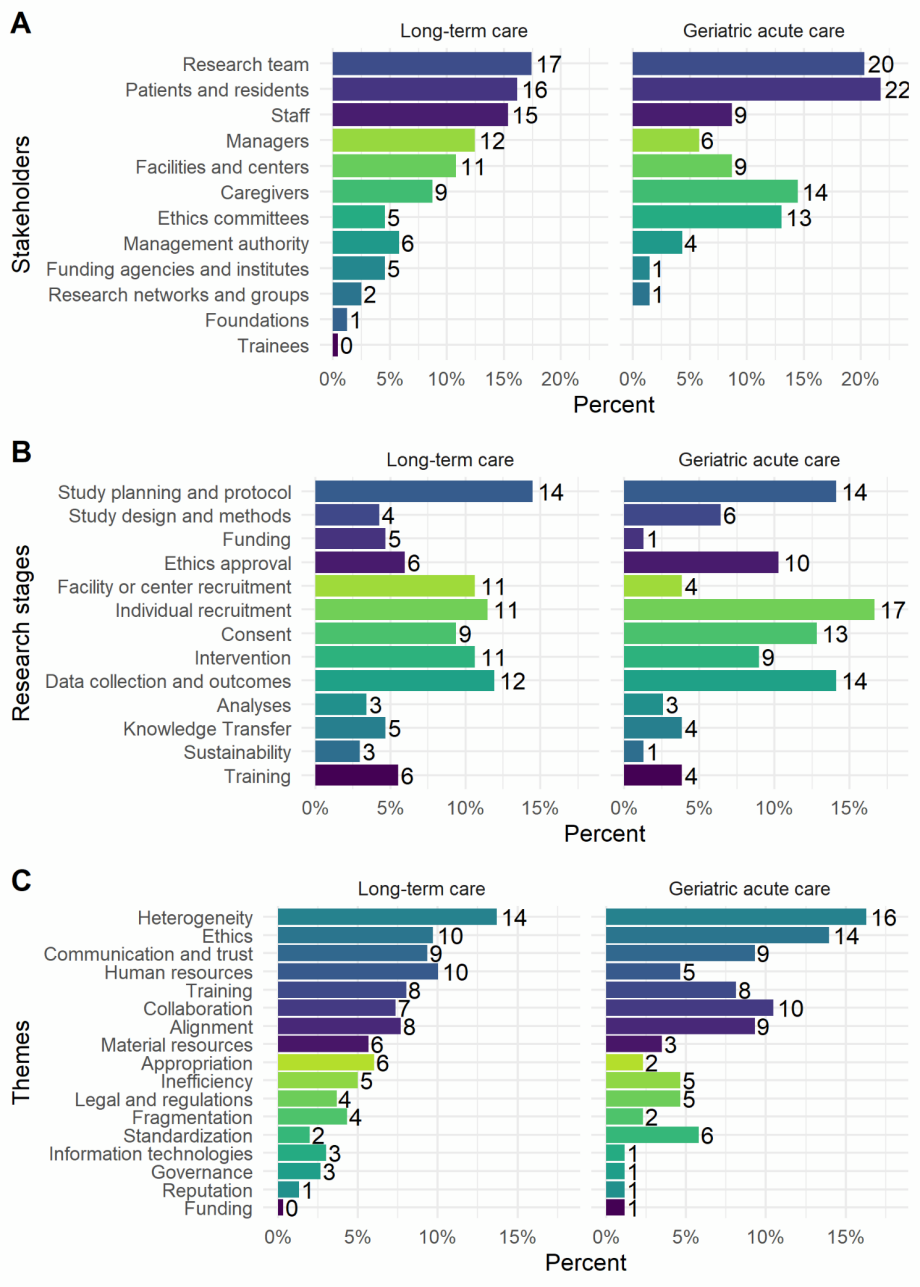
Distribution and importance of stakeholders, research stages, and themes. **Notes. (A)** Stakeholders. **(B)** Research stages. **(C)** Themes. Articles are the basic unit of analysis. Themes include content coded as barriers, facilitators, themes, or recommendations

Among the 68 articles, we quantified 1868 intersections between specific stakeholders, stages, and transversal themes, of which 668 were barriers, 268 were facilitators, 442 were broad themes, and 490 were suggestions or recommendations. With articles as the unit of quantification, Fig. 1 shows the relative importance of stakeholders, stages, and themes according to the LTC and GAC settings. Notable differences in stakeholders between settings are the greater relative importance of staff and managers in LTC, and of caregivers and ethics committees in GAC; in research stages: funding and facility recruitment in LTC, and ethics approval and individual recruitment in GAC. Themes differentially more relevant in LTC were human resources, material resources and appropriation, compared to ethics and standardization in GAC. Supplementary Fig. 3 summarizes the perception of stakeholders, stages, and themes as barriers or facilitators: ethical considerations were strongly skewed towards being barriers, whereas the themes of communication and trust, collaboration, and appropriation were preferentially perceived as facilitators.

### Quantitative synthesis: conceptual framework and maps

**Fig. 2:**
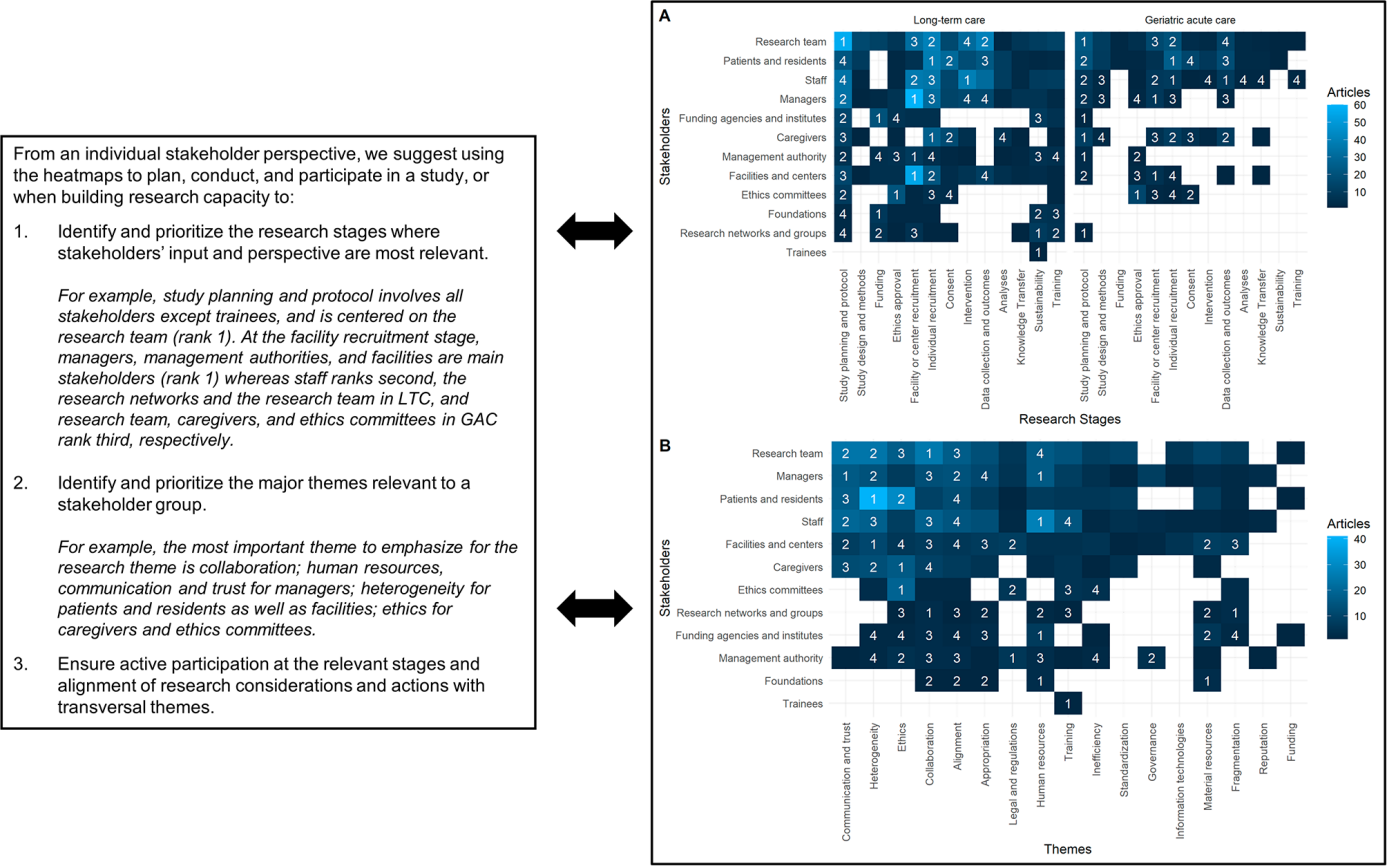
Interaction heatmaps for stakeholders by importance of research stages and transversal themes. The numbers in the heatmap cells indicate the order of importance for each stakeholder by the number of articles published. Duplicate cell ranks are due to ties. **(A)** Stakeholders by research stages. **(B)** Stakeholders by themes

**Fig. 3:**
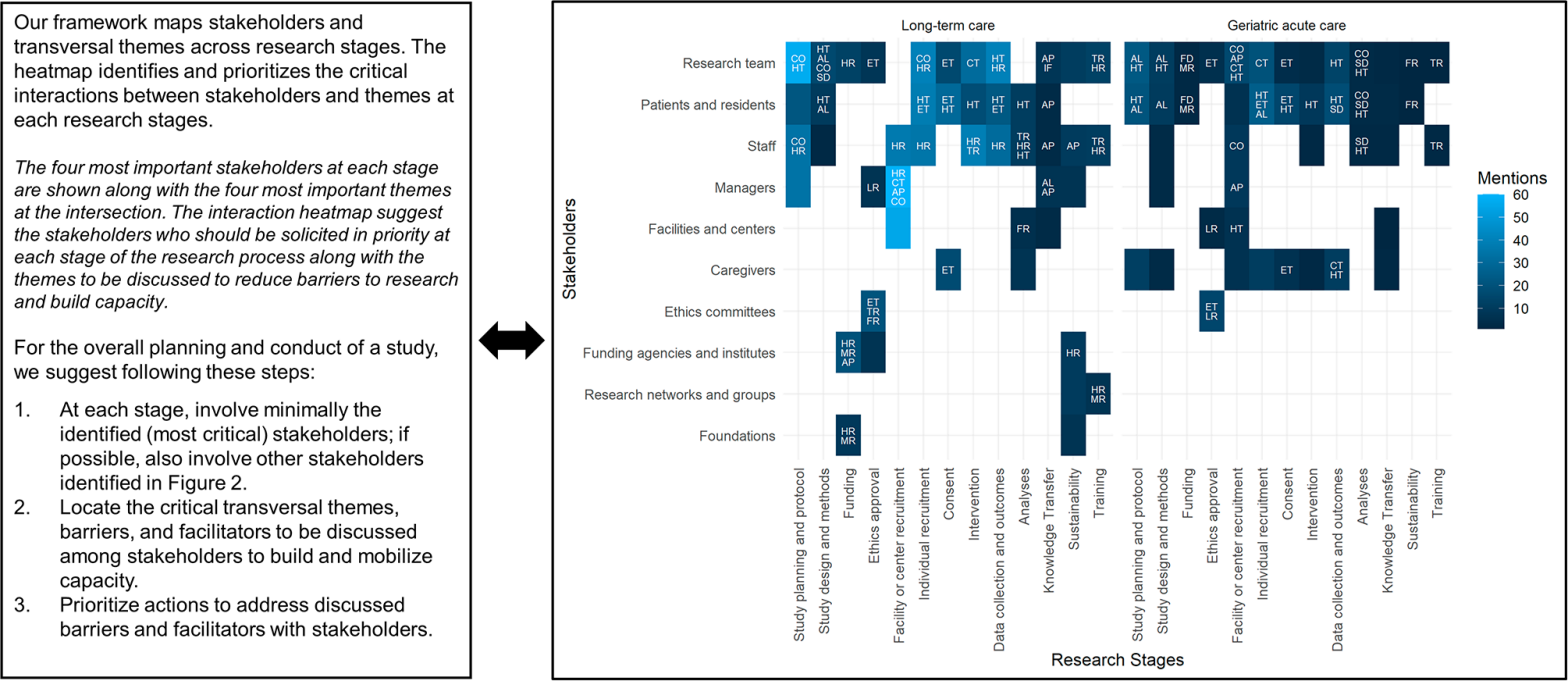
Final conceptual framework: interaction heatmap of primary stakeholders, research stages, and transversal themes, with steps to build and mobilize research capacity. AL = alignment; AP = appropriation; CT = communication and trust; CO = collaboration; ET = ethical committees; FD = funding; FR = fragmentation; HR = human resources; HT = heterogeneity; LR = legal and regulations; MR = material resources; SD = standardization; TR = training. Ties alter the exact number of themes displayed

Figures 2 and 3 synthesize intersections between stakeholders, stages, and themes, using heatmaps quantifying the density of interactions. Figure 2 A shows the relative importance of research stages for each stakeholder in LTC and GAC, as determined by the number of mentions in unique articles; Fig. 2B shows the relative relevance of themes for each stakeholder. For example, the most important themes were collaboration for the research team; communication, trust, and human resources for managers; and heterogeneity for patients and residents. Finally, Fig. 3 combines all three elements to show the most important stakeholders and themes at each research stages according to the number of mentions across all articles. To provide more context and facilitate usage of the heatmaps, Figs. 2 and 3 also propose steps to use the framework to build and mobilize research capacity.

## Discussion

In this multistep mixed methods study, we identified 12 stakeholders, 13 research stages, and 17 transversal themes involved in the research process in LTC and GAC. By quantifying their intersections in the current literature, we were able to structure and synthesize the interplay of all 42 elements using multidimensional heatmaps.

Our findings illustrate the complex and multifaceted nature of research in settings with older adults with vulnerability. We attempt to reduce this complexity by showing differences in the importance of stakeholders and themes between LTC and GAC. In LTC, staff, managers, facility recruitment, human resources (e.g., staff turnover), and material resources feature prominently [16, 17, 27, 30, 37, 38]. Conversely, in GAC, major stakeholders and themes are caregivers, ethics, individual recruitment, and standardization [14, 20, 39]. Potential underfunding and understaffing of LTC may explain why elements to ensure basic and optimal care are prioritized rather than research issues such as recruitment and standardization. Moreover, concerns with care themes in LTC rather than research themes may mirror the greater focus of clinical research on acute care settings and lack of involvement of LTC participants.

Our findings are consistent with previous reviews on barriers and underrepresentation of older adults in research to which we refer the reader for specific recommendations and potential solutions [13, 17, 19, 20, 22, 27, 39, 40]. For example, Peryer et al. identified compatibility of the intervention with current routines as the strongest factor influencing complex research process in LTC; we show that heterogeneity of patients and facilities, and alignment of research and care objectives are critical themes to facilitate research. As suggested by Mody et al., staff solicitation and communication promote individual participation; our heatmaps also reveal the importance of the interplay between the research team, staff members under the themes of collaboration, alignment, and appropriation.

By emphasizing relationships, intersections, and themes, our conceptual mapping review adds to the current body of knowledge on the topic. Beyond the differences between LTC and GAC, the variety of research questions and contexts compounds the complexity of clinical research in these settings. Our findings show that recommendations and potential solutions may not be reducible to a single list. Our heatmaps adopt a different approach to identify, prioritize, and answer three questions: who should be involved (i.e., stakeholders), when (i.e., research stages), and on what topics (i.e., themes). This may empower researchers and all stakeholders to put forth grassroots and context-specific solutions rather than predetermined ones. Our framework can be used as a blueprint to mobilize and maximize the contribution of each stakeholder at the time and on the themes where their relevance is highest. This blueprint may also serve to facilitate the training and understanding of the process of research for researchers less familiar with LTC or GAC settings.

As we used the published literature as the basis for quantification, the heatmaps show both what has been the focus of research and what hasn’t. The whitespace in the heatmaps shows potential opportunities for future research, such as training and sustainability of research.

Our study has limitations that deserve mention. First, the initial elements of our framework were issued from the environmental scan and our subject matter expertise; a different set of interviewees may have altered the initial baseline framework. While we used purposive and convenience strategies for our participant selection, we made sure to include participants with different perspectives and experiences. Second, the final elements of our framework were subject to variability in coding at each iteration; we ensured validation at 3 time points in the process. Third, we used the intersections of content within published articles as the measure of importance; this may not completely encompass in vivo relevance. Nonetheless, our framework serves to prioritize the most critical elements at play at each stage and does not deter from including additional contributions from other stakeholders and themes.

Ultimately, the usefulness of our framework depends on its ability to help researchers and other stakeholders who will use it. We believe that it is sufficiently flexible to be applied at small and large scales, for local and international contexts. We are currently conducting a multicenter validation study and are planning a pilot clinical study across the continuum of LTC and GAC in the province of Quebec. The framework and heatmaps will serve as a blueprint for the design and execution phases. Rather than requiring the involvement of all stakeholders across all research stages, the Fig. 2A heatmap will be used to determine which intersections to prioritize. We plan to use the Figs. 2B and 3 heatmaps to solicit specific facilitators and barriers at each intersection of stage, theme, and stakeholders to better understand and address the heterogeneity of research in LTC and GAC.

## Conclusions

Clinical research in long-term care and geriatric acute care is multifaceted and involves numerous stakeholders, research stages, and transversal themes. The importance of each element varies across specific care settings and research stage. Our conceptual mapping framework provides a scaffolding to address this complexity and empower stakeholders to improve each stage of the research process.

### Electronic supplementary material

Below is the link to the electronic supplementary material.

Supplementary Material 1

## Data Availability

The datasets generated and analyzed during the current study are not publicly available due to confidentiality issues but are available from the corresponding author on reasonable request.
