# Supplementary material for: Systematic review of research barriers, facilitators, and stakeholders in long-term care and geriatric settings, and a conceptual mapping framework to build research capacity
Source: BMC Geriatr. 2023 Oct 4;23:622. doi: 10.1186/s12877-023-04318-x (PMC10552295; doi:10.1186/s12877-023-04318-x)
Supplement: Supplementary file 1 — Supplementary Material 1 [file 12877_2023_4318_MOESM1_ESM.docx]

# Supplementary material

Supplemental material for:

**Systematic review of research barriers, facilitators, and stakeholders in long-term care and geriatric settings, and a conceptual mapping framework to build research capacity**

# Contents

[Supplementary methods 3](#_Toc13704)

[Search databases 3](#_Toc26097)

[Search queries 4](#_Toc2957)

[Medline [OVID] 4](#_Toc22695)

[Embase [OVID] 5](#_Toc20540)

[EBM Reviews [OVID] 6](#_Toc17087)

[PsycInfo [OVID] 6](#_Toc15777)

[CINAHL COMPLETE [EBSCO] 8](#_Toc31532)

[PubMed 11](#_Toc1961)

[Google Scholar 12](#_Toc25539)

[Worldcat 13](#_Toc6129)

[Networked Digital Library of Theses and Dissertation (NDLT) 14](#_Toc31333)

[Health Systems Evidence 14](#_Toc3263)

[National institute for Health and Care Excellence (NICE) 14](#_Toc17535)

[University of York (CRD) | Centre for Reviews and Dissemination 15](#_Toc15624)

[Prospero 15](#_Toc28549)

[Organisation for Economic Co-operation and Development (OECD) 15](#_Toc20855)

[World Health organization (WHO) 15](#_Toc5717)

[Current Gerontology and Geriatrics Research retrieved from Directory of Open Access Journals (DOAJ) 15](#_Toc17099)

[Google 16](#_Toc23208)

[Supplementary Tables 17](#_Toc20488)

[Supplementary Figures 28](#_Toc21086)

## Supplementary methods

## Search databases

The following electronic databases were searched:

- MEDLINE (Ovid)
- EMBASE (Ovid)
- CINAHL (EBSCOhost)
- PsycINFO (Ovid)
- EBM Reviews - Cochrane Database of Systematic Reviews 2005 to September 29, 2021, Database Field Guide
- ACP Journal Club 1991 to August 2021
- Database of Abstracts of Reviews of Effects 1st Quarter 2016
- Cochrane Clinical Answers September 2021
- Cochrane Central Register of Controlled Trials August 2021
- Cochrane Methodology Register 3rd Quarter 2012
- Health Technology Assessment 4th Quarter 2016
- NHS Economic Evaluation Database 1st Quarter 2016. Pubmed was searched from September 20, 2021, to September 27, 2021
- Theses on WorldCat (<https://www.worldcat.org/>) and Networked Digital Library of Theses and Dissertation (<http://search.ndltd.org/>)
- Health Systems Evidence (<http://www.healthsystemsevidence.org>)
- National Institute for Health and Care Excellence (<http://www.evidence.nhs.uk/>)
- Centre for Reviews and Dissemination (<https://www.crd.york.ac.uk/CRDWeb/>)
- Prospero (<https://www.crd.york.ac.uk/prospero/>)
- Organisation for Economic Co-operation and Development (<https://data.oecd.org/>)
- World Health Organization (<https://www.who.int/home/search>)
- Directory of Open Access Journals (https://www.doaj.org/), specifically Current Gerontology and Geriatrics Research (<https://www.hindawi.com/journals/cggr/>)
- Google Scholar with the first 20 results screened.

The Medline strategy was peer reviewed by another senior information specialist prior to execution using the PRESS Checklist (3). Duplicates were removed in EndNote using the method reported by W. Bramer (4).

### Search queries

##### Medline [OVID]

Ovid MEDLINE(R) and Epub Ahead of Print, In-Process, In-Data-Review & Other Non-Indexed Citations, Daily and Versions(R)

| **#** | **Searches** | **Results** |
| --- | --- | --- |
| 1 | exp Geriatrics/ | 30706 |
| 2 | exp Geriatric nursing/ | 13756 |
| 3 | exp Health Services for the Aged/ | 18071 |
| 4 | exp Long-Term Care/ | 27020 |
| 5 | exp Nursing Homes/ | 41738 |
| 6 | residential facilities/ or homes for the aged/ | 19916 |
| 7 | exp Skilled Nursing Facilities/ | 4785 |
| 8 | ((long term care or chronic care or residential or nursing or old or older or aged or elder* or senior* or assisted living) adj2 (facilit* or home* or residence* or center* or centre* or hospital*)).tw,kf. | 72852 |
| 9 | ((health service* or department* or unit* or ward*) adj2 (elder* or old or older or aged or senior* or long term care or chronic care)).tw,kf. | 3471 |
| 10 | (centre* hospitalier* de soin* de longue duree or CHSLD or heberg*).tw,kf. | 38 |
| 11 | (Aged/ or frail elderly/) and (Hospitals/ or hospitalization/) | 57440 |
| 12 | ((acute or short) adj2 care adj2 (elder* or old or older or aged or senior*)).tw,kf. | 331 |
| 13 | (((unite or soins) adj2 courte duree) and (geriatri* or aine*)).tw,kf. | 2 |
| 14 | (geriatric* or gerontolo*).tw,kf. | 75652 |
| 15 | or/1-14 | 256537 |
| 16 | research*.ti,kf. | 363827 |
| 17 | 15 and 16 | 5527 |
| 18 | (barrier* or obstacle* or challenge* or issue* or difficult* or problem* or constraint* or hindrance* or negative* or drawback* or liabilit* or inconvenience* or block* or prevent* or hinder* or limit*).tw,kf. | 7577302 |
| 19 | ((patient* or subject* or volunteer* or geriatric* or old or older or elder* or senior* or aged) adj2 (recruit* or select* or attrition or dropout or drop out or participat*)).tw,kf. | 238670 |
| 20 | 18 or 19 | 7728294 |
| 21 | 17 and 20 | 2348 |
| 22 | (Facilitat* or enabl* or favor* or simplif* or encourag* or stimulat* or promot* or "giv* rise to" or "gav* rise to" or "result* in" or initiat* or "bring forth*" or "brought forth" or benefit* or boost* or increase* or spur* or inspir* or propel* or increas* or augment* or fuel* or incit* or motivat* *).tw,kf. | 10488845 |
| 23 | 17 and 22 | 2229 |
| 24 | (Solution* or strateg* or answer* or resolv* or solv* or fix* or correct* or address* or remediat*).tw,kf. | 4013798 |
| 25 | 17 and 24 | 1150 |
| 26 | 21 or 23 or 25 | 3414 |
| 27 | (exp child/ or exp infant/ or adolescent/) not exp adult/ | 1978939 |
| 28 | (newborn* or new-born* or neonat* or neo-nat* or infan* or child* or adolesc* or paediatr* or pediatr* or baby* or babies* or toddler* or kid or kids or boy* or girl* or juvenile* or teen* or youth* or pubescen* or preadolesc* or prepubesc* or preteen or tween).ti. | 1598847 |
| 29 | (pediatr* or paediatr*).jw. | 600247 |
| 30 | 27 or 28 or 29 | 2594195 |
| 31 | 26 not 30 | 3336 |

#####

##### Embase [OVID]

| **#** | **Searches** | **Results** |
| --- | --- | --- |
| 1 | Geriatrics/ or geriatric ward/ | 31656 |
| 2 | Geriatric nursing/ | 12284 |
| 3 | elderly care/ | 41284 |
| 4 | home for the aged/ | 11255 |
| 5 | long term care/ | 134820 |
| 6 | nursing home/ | 55651 |
| 7 | Residential home/ | 7400 |
| 8 | ((long term care or chronic care or residential or nursing or old or older or aged or elder* or senior* or assisted living) adj2 (facilit* or home* or residence* or center* or centre* or hospital* or institution*)).tw,kw. | 99372 |
| 9 | ((health service* or department* or unit* or ward*) adj2 (elder* or old or older or aged or senior* or long term care or chronic care)).tw,kw. | 5422 |
| 10 | (centre* hospitalier* de soin* de longue duree or CHSLD or heberg*).tw,kw. | 16 |
| 11 | (Aged/ or frail elderly/) and (Hospitals/ or hospitalization/) | 119476 |
| 12 | ((acute or short) adj2 care adj2 (elder* or old or older or aged or senior*)).tw,kw. | 521 |
| 13 | (((unite or soins) adj2 courte duree) and (geriatri* or aine*)).tw,kw. | 0 |
| 14 | (geriatric* or gerontolo*).tw,kw. | 97080 |
| 15 | or/1-14 | 483913 |
| 16 | research*.ti,kw. | 309675 |
| 17 | 15 and 16 | 5029 |
| 18 | (barrier* or obstacle* or challenge* or issue* or difficult* or problem* or constraint* or hindrance* or negative* or drawback* or liabilit* or inconvenience* or block* or prevent* or hinder* or limit*).tw,kw. | 9635419 |
| 19 | ((patient* or subject* or volunteer* or geriatric* or old or older or elder* or senior* or aged) adj2 (recruit* or select* or attrition or dropout or drop out or participat*)).tw,kw. | 381942 |
| 20 | 18 or 19 | 9868760 |
| 21 | 17 and 20 | 2262 |
| 22 | (Facilitat* or enabl* or favor* or simplif* or encourag* or stimulat* or promot* or "giv* rise to" or "gav* rise to" or "result* in" or initiat* or "bring forth*" or "brought forth" or benefit* or boost* or increase* or spur* or inspir* or propel* or increas* or augment* or fuel* or incit* or motivat* *).tw,kw. | 13239713 |
| 23 | 17 and 22 | 2071 |
| 24 | (Solution* or strateg* or answer* or resolv* or solv* or fix* or correct* or address* or remediat*).tw,kw. | 4944811 |
| 25 | 17 and 24 | 1152 |
| 26 | 21 or 23 or 25 | 3253 |
| 27 | (exp child/ or exp adolescent/) not exp adult/ | 2270766 |
| 28 | (newborn* or new-born* or neonat* or neo-nat* or infan* or child* or adolesc* or paediatr* or pediatr* or baby* or babies* or toddler* or kid or kids or boy* or girl* or juvenile* or teen* or youth* or pubescen* or preadolesc* or prepubesc* or preteen or tween).ti. | 1847999 |
| 29 | (pediatr* or paediatr*).jx. | 714974 |
| 30 | 27 or 28 or 29 | 2998060 |
| 31 | 26 not 30 | 3146 |
| 32 | limit 31 to embase | 1711 |

#####

##### EBM Reviews [OVID]

All EBM Reviews - Cochrane DSR, ACP Journal Club, DARE, CCA, CCTR, CMR, HTA, and NHSEED

| **#** | **Searches** | **Results** |
| --- | --- | --- |
| 1 | (Geriatric* or Gerontolo* or Home for the aged or Long term care or Nursing home* or Health Services for the Aged or Institutionalization).ti,hw. | 17404 |
| 2 | ((chronic care or old or older or aged or elder* or senior* or nursing or residential or assisted living or skilled nursing) adj2 (facilit* or home* or residence* or center* or centre* or hospital* or institution*)).ti,hw. | 7483 |
| 3 | ((health service* or department* or unit* or ward*) adj2 (elder* or old or older or aged or geriatric* or senior* or long term care or chronic care)).ti,hw. | 1177 |
| 4 | (centre* hospitalier* de soin* de longue duree or CHSLD or heberg*).ti,hw. | 0 |
| 5 | ((acute or short) adj2 care adj2 (elder* or old or older or aged or geriatric* or senior*)).ti,hw. | 84 |
| 6 | or/1-5 | 21011 |
| 7 | research*.ti,hw. | 52934 |
| 8 | 6 and 7 | 1514 |
| 9 | (barrier* or obstacle* or challenge* or issue* or difficult* or problem* or constraint* or hindrance* or negative* or drawback* or liabilit* or inconvenience* or block* or prevent* or hinder* or limit*).ti,hw. | 194450 |
| 10 | ((patient* or subject* or volunteer* or geriatric* or old or older or elder* or senior* or aged) adj2 (recruit* or select* or attrition or dropout or drop out or participat*)).ti,hw. | 12187 |
| 11 | 9 or 10 | 204822 |
| 12 | 8 and 11 | 342 |
| 13 | (Facilitat* or enabl* or favor* or simplif* or encourag* or stimulat* or promot* or "giv* rise to" or "gav* rise to" or "result* in" or initiat* or "bring forth*" or "brought forth" or benefit* or boost* or increase* or spur* or inspir* or propel* or increas* or augment* or fuel* or incit* or motivat* *).ti,hw. | 227997 |
| 14 | 8 and 13 | 319 |
| 15 | (Solution* or strateg* or answer* or resolv* or solv* or fix* or correct* or address* or remediat*).ti,hw. | 56533 |
| 16 | 8 and 15 | 55 |
| 17 | 12 or 14 or 16 | 590 |
| 18 | remove duplicates from 17 | 586 |

##### PsycInfo [OVID]

| **#** | **Searches** | **Results** |
| --- | --- | --- |
| 1 | exp Geriatrics/ | 14375 |
| 2 | exp Geriatric Patients/ | 13785 |
| 3 | exp gerontology/ | 9857 |
| 4 | exp Elder Care/ | 5263 |
| 5 | exp long term care/ | 5658 |
| 6 | exp nursing homes/ | 9362 |
| 7 | exp nursing home residents/ | 2649 |
| 8 | exp institutionalization/ or exp Residential Care Institutions/ | 76715 |
| 9 | ((long term care or chronic care or residential or nursing or old or older or aged or elder* or senior* or assisted living) adj2 (facilit* or home* or residence* or center* or centre* or hospital*)).tw,hw. | 30584 |
| 10 | ((health service* or department* or unit* or ward*) adj2 (elder* or old or older or aged or senior* or long term care or chronic care)).tw,hw. | 1397 |
| 11 | (centre* hospitalier* de soin* de longue duree or CHSLD or heberg*).tw,hw. | 19 |
| 12 | (Hospitals/ or Hospitalized Patients/ or Hospitalization/) and (Aging/ or Older Adulthood/) | 615 |
| 13 | ((acute or short) adj2 care adj2 (elder* or old or older or aged or senior*)).tw,hw. | 117 |
| 14 | (((unite or soins) adj2 courte duree) and (geriatri* or aine*)).tw,hw. | 1 |
| 15 | (geriatric* or gerontolo*).tw,hw. | 46492 |
| 16 | or/1-15 | 137149 |
| 17 | research*.ti,hw. | 117443 |
| 18 | 16 and 17 | 2549 |
| 19 | (barrier* or obstacle* or challenge* or issue* or difficult* or problem* or constraint* or hindrance* or negative* or drawback* or liabilit* or inconvenience* or block* or prevent* or hinder* or limit*).tw,hw. | 2020686 |
| 20 | ((patient* or subject* or volunteer* or geriatric* or old or older or elder* or senior* or aged) adj2 (recruit* or select* or attrition or dropout or drop out or participat*)).tw,hw. | 33810 |
| 21 | 19 or 20 | 2039529 |
| 22 | 18 and 21 | 1514 |
| 23 | (Facilitat* or enabl* or favor* or simplif* or encourag* or stimulat* or promot* or "giv* rise to" or "gav* rise to" or "result* in" or initiat* or "bring forth*" or "brought forth" or benefit* or boost* or increase* or spur* or inspir* or propel* or increas* or augment* or fuel* or incit* or motivat* *).tw,hw. | 1808846 |
| 24 | 18 and 23 | 1083 |
| 25 | (Solution* or strateg* or answer* or resolv* or solv* or fix* or correct* or address* or remediat*).tw,hw. | 1049542 |
| 26 | 18 and 25 | 770 |
| 27 | 22 or 24 or 26 | 2027 |
| 28 | (newborn* or new-born* or neonat* or neo-nat* or infan* or child* or adolesc* or paediatr* or pediatr* or baby* or babies* or toddler* or kid or kids or boy* or girl* or juvenile* or teen* or youth* or pubescen* or preadolesc* or prepubesc* or preteen or tween).ti. | 594442 |
| 29 | (pediatr* or paediatr*).jn. | 4118 |
| 30 | 28 or 29 | 595595 |
| 31 | 27 not 30 | 1886 |

##### CINAHL COMPLETE [EBSCO]

| **Question** | **Opérateurs de restriction/Opérateurs d'expansion** | **Opérateurs de restriction/Opérateurs d'expansion** | **Dernière exécution par** | **Résultats** |
| --- | --- | --- | --- | --- |
| S1 | (MH "Long Term Care") | Opérateurs d'expansion - Appliquer des sujets équivalents Modes de recherche - Booléen/Phrase | Interface - EBSCOhost Research Databases Ecran de recherche - Recherche avancée Base de données - CINAHL | 26,785 |
| S2 | (MH "Nursing Homes") | Opérateurs d'expansion - Appliquer des sujets équivalents Modes de recherche - Booléen/Phrase | Interface - EBSCOhost Research Databases Ecran de recherche - Recherche avancée Base de données - CINAHL | 24,472 |
| S3 | (MH "Gerontologic Care") | Opérateurs d'expansion - Appliquer des sujets équivalents Modes de recherche - Booléen/Phrase | Interface - EBSCOhost Research Databases Ecran de recherche - Recherche avancée Base de données - CINAHL | 24,401 |
| S4 | (MH "Skilled Nursing Facilities") | Opérateurs d'expansion - Appliquer des sujets équivalents Modes de recherche - Booléen/Phrase | Interface - EBSCOhost Research Databases Ecran de recherche - Recherche avancée Base de données - CINAHL | 4,493 |
| S5 | (MH "Nursing Home Patients") | Opérateurs d'expansion - Appliquer des sujets équivalents Modes de recherche - Booléen/Phrase | Interface - EBSCOhost Research Databases Ecran de recherche - Recherche avancée Base de données - CINAHL | 14,505 |
| S6 | (MH "Health Services for the Aged") | Opérateurs d'expansion - Appliquer des sujets équivalents Modes de recherche - Booléen/Phrase | Interface - EBSCOhost Research Databases Ecran de recherche - Recherche avancée Base de données - CINAHL | 6,830 |
| S7 | (MH "Gerontologic Nursing") | Opérateurs d'expansion - Appliquer des sujets équivalents Modes de recherche - Booléen/Phrase | Interface - EBSCOhost Research Databases Ecran de recherche - Recherche avancée Base de données - CINAHL | 13,384 |
| S8 | (MH "Geriatrics") | Opérateurs d'expansion - Appliquer des sujets équivalents Modes de recherche - Booléen/Phrase | Interface - EBSCOhost Research Databases Ecran de recherche - Recherche avancée Base de données - CINAHL | 5,696 |
| S9 | TI ( ((long term care or chronic care or residential or nursing or old or older or aged or elder* or senior* or assisted living) N2 (facilit* or home* or residence* or center* or centre* or hospital*)) ) OR AB ( ((long term care or chronic care or residential or nursing or old or older or aged or elder* or senior* or assisted living) N2 (facilit* or home* or residence* or center* or centre* or hospital* or institution*)) ) | Opérateurs d'expansion - Appliquer des sujets équivalents Modes de recherche - Booléen/Phrase | Interface - EBSCOhost Research Databases Ecran de recherche - Recherche avancée Base de données - CINAHL | 63,134 |
| S10 | TI ( ((health service* or department* or unit* or ward*) N2 (elder* or old or older or aged or senior* or long term care or chronic care)) ) OR AB ( ((health service* or department* or unit* or ward*) N2 (elder* or old or older or aged or senior* or long term care or chronic care)) ) | Opérateurs d'expansion - Appliquer des sujets équivalents Modes de recherche - Booléen/Phrase | Interface - EBSCOhost Research Databases Ecran de recherche - Recherche avancée Base de données - CINAHL | 4,287 |
| S11 | TI ( (centre* hospitalier* de soin* de longue duree or CHSLD or heberg*) ) OR AB ( (centre* hospitalier* de soin* de longue duree or CHSLD or heberg*) ) | Opérateurs d'expansion - Appliquer des sujets équivalents Modes de recherche - Booléen/Phrase | Interface - EBSCOhost Research Databases Ecran de recherche - Recherche avancée Base de données - CINAHL | 54 |
| S12 | (MH "Aged, Hospitalized") | Opérateurs d'expansion - Appliquer des sujets équivalents Modes de recherche - Booléen/Phrase | Interface - EBSCOhost Research Databases Ecran de recherche - Recherche avancée Base de données - CINAHL | 3,900 |
| S13 | TI ( ((acute or short) N2 care N2 (elder* or old or older or aged or senior*)) ) OR AB ( ((acute or short) N2 care N2(elder* or old or older or aged or senior*)) ) | Opérateurs d'expansion - Appliquer des sujets équivalents Modes de recherche - Booléen/Phrase | Interface - EBSCOhost Research Databases Ecran de recherche - Recherche avancée Base de données - CINAHL | 656 |
| S14 | TI ( (((unite or soins) N2 courte duree) and (geriatri* or aine*)) ) OR AB ( (((unite or soins) N2 courte duree) and (geriatri* or aine*)) ) | Opérateurs d'expansion - Appliquer des sujets équivalents Modes de recherche - Booléen/Phrase | Interface - EBSCOhost Research Databases Ecran de recherche - Recherche avancée Base de données - CINAHL | 1 |
| S15 | TI ( (geriatric* or gerontolo*) ) OR AB ( (geriatric* or gerontolo*) ) | Opérateurs d'expansion - Appliquer des sujets équivalents Modes de recherche - Booléen/Phrase | Interface - EBSCOhost Research Databases Ecran de recherche - Recherche avancée Base de données - CINAHL | 31,125 |
| S16 | TI research* | Opérateurs d'expansion - Appliquer des sujets équivalents Modes de recherche - Booléen/Phrase | Interface - EBSCOhost Research Databases Ecran de recherche - Recherche avancée Base de données - CINAHL | 138,384 |
| S17 | TI ( (barrier* or obstacle* or challenge* or issue* or difficult* or problem* or constraint* or hindrance* or negative* or drawback* or liabilit* or inconvenience* or block* or prevent* or hinder* or limit*) ) OR AB ( (barrier* or obstacle* or challenge* or issue* or difficult* or problem* or constraint* or hindrance* or negative* or drawback* or liabilit* or inconvenience* or block* or prevent* or hinder* or limit*) ) | Opérateurs d'expansion - Appliquer des sujets équivalents Modes de recherche - Booléen/Phrase | Interface - EBSCOhost Research Databases Ecran de recherche - Recherche avancée Base de données - CINAHL | 1,690,366 |
| S18 | TI ( ((patient* or subject* or volunteer* or geriatric* or old or older or elder* or senior* or aged) N2 (recruit* or select* or attrition or dropout or drop out or participat*)) ) OR AB ( ((patient* or subject* or volunteer* or geriatric* or old or older or elder* or senior* or aged) N2 (recruit* or select* or attrition or dropout or drop out or participat*)) ) | Opérateurs d'expansion - Appliquer des sujets équivalents Modes de recherche - Booléen/Phrase | Interface - EBSCOhost Research Databases Ecran de recherche - Recherche avancée Base de données - CINAHL | 85,398 |
| S19 | S17 OR S18 | Opérateurs d'expansion - Appliquer des sujets équivalents Modes de recherche - Booléen/Phrase | Interface - EBSCOhost Research Databases Ecran de recherche - Recherche avancée Base de données - CINAHL | 1,741,559 |
| S20 | TI ( (Facilitat* or enabl* or favor* or simplif* or encourag* or stimulat* or promot* or "giv* rise to" or "gav* rise to" or "result* in" or initiat* or "bring forth*" or "brought forth" or benefit* or boost* or increase* or spur* or inspir* or propel* or increas* or augment* or fuel* or incit* or motivat* *) ) OR AB ( (Facilitat* or enabl* or favor* or simplif* or encourag* or stimulat* or promot* or "giv* rise to" or "gav* rise to" or "result* in" or initiat* or "bring forth*" or "brought forth" or benefit* or boost* or increase* or spur* or inspir* or propel* or increas* or augment* or fuel* or incit* or motivat* *) ) | Opérateurs d'expansion - Appliquer des sujets équivalents Modes de recherche - Booléen/Phrase | Interface - EBSCOhost Research Databases Ecran de recherche - Recherche avancée Base de données - CINAHL | 2,569,285 |
| S21 | TI ( (Solution* or strateg* or answer* or resolv* or solv* or fix* or correct* or address* or remediat*) ) OR AB ( (Solution* or strateg* or answer* or resolv* or solv* or fix* or correct* or address* or remediat*) ) | Opérateurs d'expansion - Appliquer des sujets équivalents Modes de recherche - Booléen/Phrase | Interface - EBSCOhost Research Databases Ecran de recherche - Recherche avancée Base de données - CINAHL | 816,819 |
| S22 | S1 OR S2 OR S3 OR S4 OR S5 OR S6 OR S7 OR S8 OR S9 OR S10 OR S11 OR S12 OR S13 OR S14 OR S15 | Opérateurs d'expansion - Appliquer des sujets équivalents Modes de recherche - Booléen/Phrase | Interface - EBSCOhost Research Databases Ecran de recherche - Recherche avancée Base de données - CINAHL | 152,716 |
| S23 | S16 AND S22 | Opérateurs d'expansion - Appliquer des sujets équivalents Modes de recherche - Booléen/Phrase | Interface - EBSCOhost Research Databases Ecran de recherche - Recherche avancée Base de données - CINAHL | 3,117 |
| S24 | S19 AND S23 | Opérateurs d'expansion - Appliquer des sujets équivalents Modes de recherche - Booléen/Phrase | Interface - EBSCOhost Research Databases Ecran de recherche - Recherche avancée Base de données - CINAHL | 1,157 |
| S25 | S20 AND S23 | Opérateurs d'expansion - Appliquer des sujets équivalents Modes de recherche - Booléen/Phrase | Interface - EBSCOhost Research Databases Ecran de recherche - Recherche avancée Base de données - CINAHL | 1,203 |
| S26 | S21 AND S23 | Opérateurs d'expansion - Appliquer des sujets équivalents Modes de recherche - Booléen/Phrase | Interface - EBSCOhost Research Databases Ecran de recherche - Recherche avancée Base de données - CINAHL | 591 |
| S27 | (S24 OR S25 OR S26) | Opérateurs d'expansion - Appliquer des sujets équivalents Modes de recherche - Booléen/Phrase | Interface - EBSCOhost Research Databases Ecran de recherche - Recherche avancée Base de données - CINAHL | 1,752 |

##### PubMed

| **Search** |  |
| --- | --- |
| ((((((((Geriatric*[ti] OR Geriatric*[mh]) OR (Gerontolo*[ti] OR Gerontolo*[mh]) OR ("Home for the aged"[ti] OR "Home for the aged"[mh]) OR ("Long term care"[ti] OR "Long term care"[mh]) OR (Nursing home*[ti] OR Nursing home*[mh]) OR ("Health Services for the Aged"[ti] OR "Health Services for the Aged"[mh]) OR ("Institutionalization"[ti] OR "Institutionalization"[mh]))) OR ((((health service*[ti] OR health service*[mh]) OR (department*[ti] OR department*[mh]) OR (unit*[ti] OR unit*[mh]) OR (ward*[ti] OR ward*[mh])) AND ((elder*[ti] OR elder*[mh]) OR ("old"[ti] OR "old"[mh]) OR ("older"[ti] OR "older"[mh]) OR ("aged"[ti] OR "aged"[mh]) OR (geriatric*[ti] OR geriatric*[mh]) OR (senior*[ti] OR senior*[mh]) OR ("long term care"[ti] OR "long term care"[mh]) OR ("chronic care"[ti] OR "chronic care"[mh]))))) OR (((centre* hospitalier* de soin* de longue duree[ti] OR centre* hospitalier* de soin* de longue duree[mh]) OR ("CHSLD"[ti] OR "CHSLD"[mh]) OR (heberg*[ti] OR heberg*[mh])))) OR (((("acute"[ti] OR "acute"[mh]) OR ("short"[ti] OR "short"[mh])) AND ("care"[ti] OR "care"[mh]) AND ((elder*[ti] OR elder*[mh]) OR ("old"[ti] OR "old"[mh]) OR ("older"[ti] OR "older"[mh]) OR ("aged"[ti] OR "aged"[mh]) OR (geriatric*[ti] OR geriatric*[mh]) OR (senior*[ti] OR senior*[mh]))))) OR (((("chronic care"[ti] OR "chronic care"[mh]) OR ("old"[ti] OR "old"[mh]) OR ("older"[ti] OR "older"[mh]) OR ("aged"[ti] OR "aged"[mh]) OR (elder*[ti] OR elder*[mh]) OR (senior*[ti] OR senior*[mh]) OR ("nursing"[ti] OR "nursing"[mh]) OR ("residential"[ti] OR "residential"[mh]) OR ("assisted living"[ti] OR "assisted living"[mh]) OR ("skilled nursing"[ti] OR "skilled nursing"[mh])) AND ((facilit*[ti] OR facilit*[mh]) OR (home*[ti] OR home*[mh]) OR (residence*[ti] OR residence*[mh]) OR (center*[ti] OR center*[mh]) OR (centre*[ti] OR centre*[mh]) OR (hospital*[ti] OR hospital*[mh]) OR (institution*[ti] OR institution*[mh]))))) AND ((research*[ti] OR research*[mh]))) AND ((Facilitat*[ti] OR enabl*[ti] OR favor*[ti] OR simplif*[ti] OR encourag*[ti] OR stimulat*[ti] OR promot*[ti] OR giv* rise to[ti] OR gav* rise to[ti] OR result* in[ti] OR initiat*[ti] OR bring forth*[ti] OR "brought forth"[ti] OR benefit*[ti] OR boost*[ti] OR increase*[ti] OR spur*[ti] OR inspir*[ti] OR propel*[ti] OR increas*[ti] OR augment*[ti] OR fuel*[ti] OR incit*[ti] OR motivat* *[ti])) AND (2021/09/20:3000/12/31[Date - Create]) **OR** | 0 |
| ((((((((Geriatric*[ti] OR Geriatric*[mh]) OR (Gerontolo*[ti] OR Gerontolo*[mh]) OR ("Home for the aged"[ti] OR "Home for the aged"[mh]) OR ("Long term care"[ti] OR "Long term care"[mh]) OR (Nursing home*[ti] OR Nursing home*[mh]) OR ("Health Services for the Aged"[ti] OR "Health Services for the Aged"[mh]) OR ("Institutionalization"[ti] OR "Institutionalization"[mh]))) OR ((((health service*[ti] OR health service*[mh]) OR (department*[ti] OR department*[mh]) OR (unit*[ti] OR unit*[mh]) OR (ward*[ti] OR ward*[mh])) AND ((elder*[ti] OR elder*[mh]) OR ("old"[ti] OR "old"[mh]) OR ("older"[ti] OR "older"[mh]) OR ("aged"[ti] OR "aged"[mh]) OR (geriatric*[ti] OR geriatric*[mh]) OR (senior*[ti] OR senior*[mh]) OR ("long term care"[ti] OR "long term care"[mh]) OR ("chronic care"[ti] OR "chronic care"[mh]))))) OR (((centre* hospitalier* de soin* de longue duree[ti] OR centre* hospitalier* de soin* de longue duree[mh]) OR ("CHSLD"[ti] OR "CHSLD"[mh]) OR (heberg*[ti] OR heberg*[mh])))) OR (((("acute"[ti] OR "acute"[mh]) OR ("short"[ti] OR "short"[mh])) AND ("care"[ti] OR "care"[mh]) AND ((elder*[ti] OR elder*[mh]) OR ("old"[ti] OR "old"[mh]) OR ("older"[ti] OR "older"[mh]) OR ("aged"[ti] OR "aged"[mh]) OR (geriatric*[ti] OR geriatric*[mh]) OR (senior*[ti] OR senior*[mh]))))) OR (((("chronic care"[ti] OR "chronic care"[mh]) OR ("old"[ti] OR "old"[mh]) OR ("older"[ti] OR "older"[mh]) OR ("aged"[ti] OR "aged"[mh]) OR (elder*[ti] OR elder*[mh]) OR (senior*[ti] OR senior*[mh]) OR ("nursing"[ti] OR "nursing"[mh]) OR ("residential"[ti] OR "residential"[mh]) OR ("assisted living"[ti] OR "assisted living"[mh]) OR ("skilled nursing"[ti] OR "skilled nursing"[mh])) AND ((facilit*[ti] OR facilit*[mh]) OR (home*[ti] OR home*[mh]) OR (residence*[ti] OR residence*[mh]) OR (center*[ti] OR center*[mh]) OR (centre*[ti] OR centre*[mh]) OR (hospital*[ti] OR hospital*[mh]) OR (institution*[ti] OR institution*[mh]))))) AND ((research*[ti] OR research*[mh]))) AND (((barrier*[ti] OR obstacle*[ti] OR challenge*[ti] OR issue*[ti] OR difficult*[ti] OR problem*[ti] OR constraint*[ti] OR hindrance*[ti] OR negative*[ti] OR drawback*[ti] OR liabilit*[ti] OR inconvenience*[ti] OR block*[ti] OR prevent*[ti] OR hinder*[ti] OR limit*[ti])) OR (((patient*[ti] OR subject*[ti] OR volunteer*[ti] OR geriatric*[ti] OR "old"[ti] OR "older"[ti] OR elder*[ti] OR senior*[ti] OR "aged"[ti]) AND (recruit*[ti] OR select*[ti] OR "attrition"[ti] OR "dropout"[ti] OR "drop out"[ti] OR participat*[ti])))) AND (2021/09/20:3000/12/31[Date - Create]) **OR** | 1 |
| ((((((((Geriatric*[ti] OR Geriatric*[mh]) OR (Gerontolo*[ti] OR Gerontolo*[mh]) OR ("Home for the aged"[ti] OR "Home for the aged"[mh]) OR ("Long term care"[ti] OR "Long term care"[mh]) OR (Nursing home*[ti] OR Nursing home*[mh]) OR ("Health Services for the Aged"[ti] OR "Health Services for the Aged"[mh]) OR ("Institutionalization"[ti] OR "Institutionalization"[mh]))) OR ((((health service*[ti] OR health service*[mh]) OR (department*[ti] OR department*[mh]) OR (unit*[ti] OR unit*[mh]) OR (ward*[ti] OR ward*[mh])) AND ((elder*[ti] OR elder*[mh]) OR ("old"[ti] OR "old"[mh]) OR ("older"[ti] OR "older"[mh]) OR ("aged"[ti] OR "aged"[mh]) OR (geriatric*[ti] OR geriatric*[mh]) OR (senior*[ti] OR senior*[mh]) OR ("long term care"[ti] OR "long term care"[mh]) OR ("chronic care"[ti] OR "chronic care"[mh]))))) OR (((centre* hospitalier* de soin* de longue duree[ti] OR centre* hospitalier* de soin* de longue duree[mh]) OR ("CHSLD"[ti] OR "CHSLD"[mh]) OR (heberg*[ti] OR heberg*[mh])))) OR (((("acute"[ti] OR "acute"[mh]) OR ("short"[ti] OR "short"[mh])) AND ("care"[ti] OR "care"[mh]) AND ((elder*[ti] OR elder*[mh]) OR ("old"[ti] OR "old"[mh]) OR ("older"[ti] OR "older"[mh]) OR ("aged"[ti] OR "aged"[mh]) OR (geriatric*[ti] OR geriatric*[mh]) OR (senior*[ti] OR senior*[mh]))))) OR (((("chronic care"[ti] OR "chronic care"[mh]) OR ("old"[ti] OR "old"[mh]) OR ("older"[ti] OR "older"[mh]) OR ("aged"[ti] OR "aged"[mh]) OR (elder*[ti] OR elder*[mh]) OR (senior*[ti] OR senior*[mh]) OR ("nursing"[ti] OR "nursing"[mh]) OR ("residential"[ti] OR "residential"[mh]) OR ("assisted living"[ti] OR "assisted living"[mh]) OR ("skilled nursing"[ti] OR "skilled nursing"[mh])) AND ((facilit*[ti] OR facilit*[mh]) OR (home*[ti] OR home*[mh]) OR (residence*[ti] OR residence*[mh]) OR (center*[ti] OR center*[mh]) OR (centre*[ti] OR centre*[mh]) OR (hospital*[ti] OR hospital*[mh]) OR (institution*[ti] OR institution*[mh]))))) AND ((research*[ti] OR research*[mh]))) AND ((Solution*[ti] OR strateg*[ti] OR answer*[ti] OR resolv*[ti] OR solv*[ti] OR fix*[ti] OR correct*[ti] OR address*[ti] OR remediat*[ti])) AND (2021/09/20:3000/12/31[Date - Create]) | 1 |

##### Google Scholar

| **Search** | **Results** |
| --- | --- |
| Geriatric* OR Gerontolo* OR Home for the aged OR Long term care OR Nursing home*) AND research | 0 selected articles within the first 20 results |
| (centre* hospitalier* de soin* de longue duree or CHSLD or heberg*) AND Recherche | 0 selected articles within the first 20 results |
| (((acute or short) AROUND 2 care AROUND 2 (elder* or old or older or aged or geriatric* or senior*))) AND research | 0 selected articles within the first 20 results |
| (UCDG OR (unité de courte durée gériatrique)) AND recherche | 0 selected articles within the first 20 results |

##### Worldcat

| **Search** | **Results** |
| --- | --- |
| Kw: (Geriatric* OR Gerontolo* OR Home for the aged OR Long term care or Nursing home*) AND research*  Ti: (Geriatric* OR Gerontolo* OR Home for the aged OR Long term care or Nursing home*) AND research  Format: Article (117) | 1 selected article within 117 results |

##### Networked Digital Library of Theses and Dissertation (NDLT)

<http://search.ndltd.org/>

| **Search** | **Results** |
| --- | --- |
| (geriatric* OR gerontology* ) AND research  Tagged with:   - Gerontology - Health - Studies - Older - Aging - Sciences - Research - English or French | 1 selected article within 518 results |
| CHSLD AND Recherche  Tagged with:   - French | 0 selected article within 29 results |
| centre hospitalier de soins de longue durée AND recherche  Tagged with:   - Sciences - Santé - Health - French | 0 selected article within 347 results |
| UCDG AND Recherche  Tagged with:   - French | 0 selected articles out of 1 result |
| unité de courte durée gériatrique AND recherche  Tagged with:   - French | 0 selected articles within 45 results |
| “Long term care” AND research | 0 selected article of 186 results |

##### Health Systems Evidence

[http://www.healthsystemsevidence.org](http://www.healthsystemsevidence.org/)

| **Search** | **Results** |
| --- | --- |
| (Geriatric OR geronto*) AND research | 0 selected articles within 237 |
| Nursing home* and research | 0 selected articles within first 100 results |
| Long term care AND research | 0 selected articles within first 100 results |

##### National institute for Health and Care Excellence (NICE)

<http://www.evidence.nhs.uk/>

| **Search** | **Results** |
| --- | --- |
| Nursing home* research | 0 selected articles within first 100 results |
| Long term care research | 2 selected articles within first 100 results |
| Gerontology research | 0 selected articles within first 100 results |
| Geriatric Research | 2 selected articles within first 100 results |

#####

##### University of York (CRD) | Centre for Reviews and Dissemination

<http://www.crd.york.ac.uk/crdweb/SearchPage.asp>

| **Search** | **Results** |
| --- | --- |
| Gerontology AND research (any field) | 0 selected articles within 27 results |
| (geriatric):TI AND (research):TI | 0 results |
| (nursing home):TI AND (research) | 0 selected articles within 28 results |
| (long term care):TI AND (research):TI | 0 selected results out of 1 result |

##### Prospero

<https://www.crd.york.ac.uk/prospero/>

| **Search** | **Results** |
| --- | --- |
| geriatric AND research | 0 selected articles within first 100 results |
| Gerontology AND research | 0 selected articles within 270 results |
| Nursing home* AND research | 0 selected articles within first 100 results |
| Long term care AND research | 0 selected articles within first 100 results |

#####

##### Organisation for Economic Co-operation and Development (OECD)

<https://data.oecd.org/>

| **Search** | **Results** |
| --- | --- |
| geriatric | 0 results |
| gerontology | 0 results |
| Nursing homes | 0 selected articles within first 100 results |
| Long term care | 0 selected articles within first 100 results |

##### World Health organization (WHO)

<https://www.who.int/home/search>

| **Search** | **Results** |
| --- | --- |
| Geriatric AND research | 0 results |
| Gerontology AND research | 0 results |
| Gerontology AND research | 0 results |
| long term care AND research | 0 results |

##### [Current Gerontology and Geriatrics Research](https://www.hindawi.com/journals/cggr/) retrieved from [Directory of Open Access Journals](https://www.doaj.org/) (DOAJ)

| **Search** | **Results** |
| --- | --- |
| Geriatric AND research | 1 selected article within first 100 results |
| KW: Gerontology AND research | Same article selected within 203 results |
| TI: long term care AND research | 0 selected articles within 21 results |
| TI: nursing home AND research | 0 selected articles within 2 results |

##### Google

| **Search** | **Results** |
| --- | --- |
| geriatric* research | 0 selected articles within first 20 results |
| gerontology research | 0 selected articles within first 20 results |
| Nursing home* research | 1 selected articles within first 20 results |
| Long term care and research | 0 selected articles within first 20 results |

## Supplementary Tables

###### Supplementary Table 1. Initial stakeholders and research stages

| **Initial stakeholders** |
| --- |
| Researchers based in LTC and GAC |
| Researchers in general |
| Research staff |
| Residents and patients |
| Caregivers |
| Staff (professionals) |
| Staff (others) |
| Ethics review committees |
| Managers |
| Management and regional authorities (e.g., central offices) |
| Research networks and groups |
| Funding agencies and institutes |
| Foundations |
| Trainees |
| **Initial research stages** |
| Training |
| Study planning and protocol |
| Study design and methods |
| Funding |
| Ethics approval |
| Facility or center recruitment |
| Individual recruitment |
| Consent |
| Intervention |
| Data collection |
| Outcomes |
| Analyses |
| Knowledge transfer |
| Sustainability |

###### Supplementary Table 2. Articles included in systematic qualitative review and conceptual mapping framework

| **Author** | **Year** | **Title** | **Jurisdiction** | **Methodology** | **Main objective** |
| --- | --- | --- | --- | --- | --- |
| Ahouah et al. | 2019 | End-users and caregivers’ involvement in health interventional research carried out in geriatric facilities: a systematic review | France | Review | To provide a critical overview of the involvement of caregivers and end-users in interventions in these facilities, based on Rifkin’s analytical framework |
| Baier et al. | 2021 | Nursing home leaders’ perceptions of a research partnership | USA | Qualitative | To understand nursing home leader’s motivations for participating in a research study and perceptions of the process and value. |
| Berkman et al. | 2001 | Methodologic issues in conducting research on hospitalized older people | USA | Quantitative | To describe challenges in conducting research with hospitalized geriatric patients. |
| Davies et al. | 2014 | Enabling research in care homes: an evaluation of a national network of research ready care homes | UK | Mixed | To evaluate the feasibility and early impact of an initiative to increase care home participation in research. |
| Hickman et al. | 2008 | Administrators' perspectives on ethical issues in long-term care research | USA | Qualitative | To describe the perspectives of LTC administrators regarding the value of research and the ethics of human subject’s protections |
| Backhouse et al. | 2016 | Older care-home residents as collaborators or advisors in research: a systematic review | UK | Review | To determine how older care-home residents have been involved as PPI members in care-home research. |
| Black et al. | 2008 | Predictors of providing informed consent or assent for research participation in assisted living residents | USA | Quantitative | To identify factors associated with providing either informed consent or assent for research in individuals at high risk for cognitive impairment. |
| Bowling et al. | 2019 | The 5Ts: Preliminary development of a framework to support inclusion of older adults in research | USA | Qualitative | To elicit challenges to inclusion of older adults in clinical research and to develop a preliminary framework for communicating these challenges to non-geriatrics-trained researchers. |
| Brandt et al. | 2006 | Challenges in the design and conduct of a randomized study of two interventions for liquid aspiration | USA | Qualitative | To examine the key issues in the design and implementation of the first multisite, randomized behavioral trial in dysphagia in an aging population. |
| Bravo et al. | 2010 | Research with decisionally incapacitated older adults: practices of Canadian research ethics boards | Canada | Qualitative | To report the results of a study exploring the practices and perceptions of Canadian REBs regarding such protocols. |
| Chen et al. | 2018 | Applications of Minimum Data Set in long-term care research | Taiwan | Review | To introduce the development of MDS and related research using MDS, especially the Longitudinal Older Veterans (LOVE) study in Taiwan |
| Clark et al. | 2011 | A randomized trial of the impact of survey design characteristics on response rates among nursing home providers | USA | Quantitative | To assess ways to maximize the likelihood of obtaining completed questionnaires from both the DoN and the ADMIN using a nationally representative sample of U.S. nursing homes. |
| Collingridge Moore et al. | 2019 | Research, recruitment and observational data collection in care homes: lessons from the PACE study | Europe | Methods | To describe the challenges encountered in conducting the study in England to inform the design and conduct of future international research in care homes. |
| Cowdell et al. | 2008 | Engaging older people with dementia in research: myth or possibility | UK | Review | To explore a range of strategies that were used to enable older people with dementia to become actively engaged in research ‘with’ rather than ‘on’ them. |
| Cusack et al. | 2013 | Challenges and implications for biomedical research and intervention studies in older populations: insights from the ELDERMET Study | Ireland | Review | To outline the challenges and practical difficulties experienced and overcome by the ELDERMET project |
| Dewing et al. | 2007 | Participatory research a method for process consent with persons who have dementia | UK | Methods | To describe a method for consent that focuses on persons with dementia, traditionally excluded from consent and thus from research. |
| Ellwood et al. | 2018 | Recruiting care homes to a randomised controlled trial | UK | Quantitative | To describe two methods of recruiting care homes to the trial and draws out learning to inform future studies |
| Ersek et al. | 2012 | Addressing methodological challenges in implementing the nursing home pain management algorithm randomized controlled trial | USA | Methods | To describe several issues that arose in the design and conduct of a study that compared the effectiveness of pain management algorithms coupled with a comprehensive adoption program versus the effectiveness of education alone in improving evidence-based pain assessment and management practices, decreasing pain and depressive symptoms, and enhancing mobility among NH residents. |
| Faes et al. | 2007 | Methodological issues in geriatric research | Netherlands | Methods | To discuss issues important for successful recruitment and selection of subjects, the informed consent procedure and selection of appropriate research designs and research instruments in geriatric research. |
| Frank et al. | 2021 | Participation of persons with dementia and their caregivers in research | USA | Review | To address research participation by persons living with dementia (PLWD) and their care partners in two different ways: as research participants with input on outcomes studied and as engaged research partners |
| Froggatt et al. | 2021 | Public involvement in research within care homes: benefits and challenges in the APPROACH study | UK | Qualitative | To present one way in which PIR has been integrated into the design and delivery of a multisite research study based in care homes. |
| Fudge et al. | 2007 | Involving older people in health research | UK | Review | To establish the scope and extent of the involvement of older people in health research over the past 10 years, to identify reported barriers to the involvement of older people in research and to determine the impact of the involvement of older people on research and on participants. |
| Goodman et al. | 2011 | Culture, consent, costs and care homes: Enabling older people with dementia to participate in research | UK | Qualitative | To describe factors that support and inhibit recruitment and participation of people with dementia living in care homes. |
| Gustavson et al. | 2019 | Conducting clinical research in post-acute and long-term nursing home care settings: regulatory challenges | USA | Review | To expand upon previous literature that describes challenges to research in NHs1–6 and provide lessons learned when navigating the regulatory aspects of NH research. |
| Hall et al. | 2009 | Challenges to conducting research with older people living in nursing homes | UK | Qualitative | To highlight some of the methodological challenges we experienced whilst conducting a study of the perceptions of dignity of older people living in nursing homes with a view to describing the lessons learned, responses and strategies developed and recommendations for future research study design and delivery. |
| Hanson et al. | 2010 | Successful clinical trial research in nursing homes: the Improving Decision-Making Study | USA | Qualitative | To (1) describe the methods used in this randomized trial for effective nursing home and nursing home resident recruitment and retention; and (2) describe research ethics approaches to minimize harms and maximize benefits for this population. |
| Henwood et al. | 2015 | Achieving ethics approval in residential aged care research: A protective process or barrier | Australia | Review | To discuss ethics approval in residential aged care research: A protective process or barrier |
| Holden et al. | 2018 | A procedural framework to facilitate hospital-based informed consent for dementia research | USA | Methods | To develop a procedural framework for obtaining informed consent from hospitalized ADRD patients and caregivers to participate in a minimal risk care intervention |
| Hsu et al. | 2005 | Challenges of doing intervention research with the elderly Taiwanese population: example of a Tai Chi/Movement therapy | Taiwan | Review | To address the challenges of doing intervention research with an elderly population using tai chi as an intervention and to describe the strategies used to manage those challenges. |
| Hubbard et al. | 2003 | Including older people with dementia in research: challenges and strategies | UK | Review | To examine key challenges and strategies for including older people with dementia in an ethnographic study of quality of life in institutional care settings. |
| Huybretchts et al. | 2014 | Instrumental variable applications using nursing home prescribing preferences in comparative effectiveness research† | USA | Quantitative | To evaluate the validity of instrumental variable (IV) methods based on nursing home prescribing preference to mitigate such confounding, using psychotropic medications to manage behavioral problems in dementia as a case study. |
| Ilgili et al. | 2014 | Ethics in geriatric medicine research | Turkey | Review | To evaluate the research process in geriatrics from the ethical point of view. |
| Jenkins et al. | 2016 | Overcoming challenges of conducting research in nursing homes | UK | Review | To explore the process of carrying out research in nursing homes, identifying barriers and enabling factors, and making recommendations for researchers. |
| Kapp et al. | 2004 | Protecting human participants in long-term care research: The role of state law and policy | USA | Qualitative | To analyze and interweave recommendations regarding the role of state of law and public policy in protecting older persons who are or may become participants in LTC research projects |
| Kemper et al. | 2003 | Long-term care research and policy | USA | Qualitative | To provide a framework for understanding how LTC research contributes to policy and identify barriers to funding such investments |
| Kolanowski et al. | 2013 | The triple challenge of recruiting older adults with dementia and high medical acuity in skilled nursing facilities | USA | Quantitative | To describe strategies, culled from experience, for responding to several recruitment challenges in an ongoing RCT of delirium in persons with dementia. |
| Kovach et al. | 2015 | Academic research partnerships for long-term care | USA | Review | To offer some perspectives on academic research partnerships for LTC |
| Lam et al. | 2018 | Challenges of conducting research in long-term care facilities: a systematic review | Canada | Review | To identify relevant studies that report on challenges derived from first-hand experience of empirical research studies |
| Lingler et al. | 2009 | Informed consent to research in long-term care settings | USA | Review | To describe the practical implications of nurse investigators obligation to ensure informed consent among participants in LTC research. |
| Maas et al. | 2002 | Issues in conducting research in nursing homes | USA | Review | To discuss issues and challenges of ethically acceptable and rigorous research in nursing homes that are related to dependency and institutionalization of older persons and to setting, staff, and residents characteristics. Strategies for easing the challenges of nursing research in nursing homes are offered. |
| McMurdo et al. | 2011 | Improving recruitment of older people to research through good practice | UK | Review | To examine best practice on how to effectively recruit older people to clinical research, including planning, recruitment in specific settings, involving hard to target groups and engaging older people in research. |
| Mentes et al. | 2002 | Barriers and facilitators in nursing home intervention research | USA | Qualitative | To better understand the barriers and facilitators to the research process in NH. |
| Mitchell et al. | 2006 | Advanced dementia research in the nursing home: The CASCADE study | USA | Methods | To present the methodology established in the CASCADE study and to describe how the challenges specific to this research were met |
| Mody et al. | 2008 | Recruitment and Retention of Older Adults in Aging Research | USA | Review | To provide study recruitment and retention techniques and strategies to address concerns and overcome barriers to older adult participation in clinical research. |
| Mold et al. | 2008 | Methodological challenges of researching in the care home sector | UK | Qualitative | To address the challenges encountered while undertaking a feasibility study in care home |
| Morse et al. | 2005 | Ethical issues in institutional research | USA | Review | To comment on ethical issues with health care institutions with regard to research |
| Pachana et al. | 2015 | Can we do better? Researchers' experiences with ethical review boards on projects with later life as a focus | Australia and USA | Mixed | To describe researchers’ experiences in submitting ethical proposals focused on older adult populations, including studies with persons with dementia, to ethical review boards. |
| Peri et al. | 2008 | Promoting independence in residential care: Successful recruitment for a randomized controlled trial | New Zealand | Quantitative | To describe the recruitment strategy and association between facility and staff characteristics and success of resident recruitment for the PIRC trial |
| Rantz et al. | 2006 | Entrepreneurial program of research and service to improve nursing home care | USA | Methods | To demonstrate the integration of multiple goals, multiple projects with diverse foci, and multiple fundings sources to develop an entrepreneurial program of research and service to directly affect and improve the quality of care of older adults, particularly nursing home residents. |
| Robinson et al. | 2020 | Contamination in complex healthcare trials: the falls in care homes (FinCH) study experience | UK | Qualitative | To explore the potential risks of contamination bias (when members of the ‘control’ group inadvertently receive the treatment or are exposed to the intervention) in rehabilitation studies |
| Shepherd et al. | 2015 | Setting up a clinical trial in care homes: challenges encountered and recommendations for future research practice | UK | Qualitative | To draw on the experience of setting up a randomised controlled trial in care homes |
| Shepherd et al. | 2020 | How nurses can support the inclusion in research of older people who lack capacity to consent | UK | Review | To discuss some of the challenges associated with the inclusion in research of older people who lack capacity to consent, including the involvement of relatives and friends in decision-making; describes the findings of recent research and shares resources with the aim of supporting nurses to ensure that older people in their care who lack capacity can participate in research |
| Smith et al. | 2019 | Encouraging managers of care homes for older adults to participate in research | UK | Review | To note the challenges faced by the care home sector, this article considers ways in which research studies can encourage care home managers and their homes to participate in research. |
| Snyder et al. | 2001 | Challenges of implementing intervention research in persons with dementia: Example of a glider swing intervention | USA | Mixed | To address challenges of implementing intervention studies in persons with dementia. |
| Stocker et al. | 2021 | Patient and public involvement in care home research: Reflections on the how and why of involving patient and public involvement partners in qualitative data analysis and interpretation | UK | Qualitative | To provide a critical account of the ‘how’ of PPI (patient and public involvement those with a relative residing in a care home, or involved in working with or in care homes in a voluntary or professional capacity) in our collaborative qualitative data analysis. |
| Suhonen et al. | 2013 | Older people in long-term care settings as research informants: Ethical challenges | Finland | Mixed | To analyse the research ethics of the empirical studies that focus on older people in long-term care settings as research participants. |
| Tilden et al. | 2013 | Sampling challenges in nursing home research | USA | Quantitative | To compare characteristics of nursing homes that dropped from study to those that completed the study. The focus of the study was the relationship of staff communication, teamwork, and palliative/end-of-life care practices to symptom distress and other care outcomes as perceived by family members |
| Tory et al. | 2020 | The process of obtaining informed consent to research in long term care facilities (LTCFs) | USA | Quantitative | To evaluate the LTCF personnel’s assessment of resident ability to provide to clinical research, and studied potential limitations to resident autonomy. |
| Tsouvara et al. | 2015 | Lessons learned from recruiting nursing homes to a quantitative cross-sectional pilot study | UK | Mixed | To describe the barriers and challenges faced in recruiting to a recent pilot study, consider previously implemented and proposed recruitment strategies, and propose a new multi-method approach to maximising recruitment of care homes. |
| Van Ness et al. | 2012 | Efficacy and effectiveness as aspects of cluster randomized trials with nursing home residents: Methodological insights from a pneumonia prevention trial | USA | Mixed | To discuss how efficacy and effectiveness considerations have played a role in the design and conduct of the pride trial, an ongoing cluster randomized trial of an enhanced oral hygiene intervention to prevent pneumonia in nursing home residents. We also discuss methodological responses to the challenges of conducting clinical research in nursing home settings. |
| Verbeek et al. | 2020 | The living lab in ageing and long-term care: a sustainable model for translational research improving quality of life, quality of care and quality of work | Netherlands | Methods | To describe the aim of the living lab and explain the key working mechanisms of the interdisciplinary collaboration, highlighting the scientific and societal impact. We will provide a blueprint for the model, describe its business model, and discuss challenges in getting the model operational and sustainable. |
| Watson et al. | 2020 | Influencing factors that support and build aged care research capacity: Staff perspectives | Australia | Qualitative | To explore aged care staff attitudes towards research particularly the influencing factors that promote and sustain care staff participation in research in the residential aged care facility (RACF) setting. |
| White et al. | 2000 | Alzheimer's disease research in the nursing home setting | USA | Review | To provide an overview of these issues (ethical concerns to informed consent and regarding the benefits and pitfalls of research in an institutional setting) to assist medical directors and nursing home administrative staff in the appraisal of proposed Alzheimer's disease research in their institutions |
| Williams et al. | 2001 | Obtaining family consent for participation in Alzheimer's research in a Cuban-American population: Strategies to overcome the barriers | USA | Qualitative | To describe the concerns expressed by these families in regard to providing consent to participate in research for an elder with dementia and the strategies that were successful in allaying their concerns. |
| Wilson et al. | 2011 | The value of reflexivity in resolving ethical dilemmas research in care homes | UK | Qualitative | To highlight ethical dilemmas experienced by researchers when undertaking qualitative inquiry with vulnerable older people in care homes. |
| Witham et al. | 2017 | Conducting and reporting trials for older people | UK | Qualitative | To provide practical advice on how to report randomised controlled trials that are targeted at older people. |
| Wood et al. | 2013 | Consent, including advanced consent, of older adults to research in care homes: a qualitative study of stakeholders’ views in South Wales | UK | Qualitative | To explore take holders’ views about the ethical and practical challenges associated with recruiting care home residents into research studies. |
| Zermansky et al. | 2007 | Striving to recruit: the difficulties of conducting clinical research on elderly care home residents | UK | Mixed | To highlight the issues and pitfalls of large randomized controlled trial on care home residents |

###### Supplementary Table 3. Characteristics of articles included in systematic mapping review

| **Year (n = 68)** | |
| --- | --- |
| 2000-2004 | 9 (13%) |
| 2005-2009 | 17 (25%) |
| 2010-2014 | 16 (24%) |
| 2015-2019 | 17 (25%) |
| 2020-2021 | 9 (13%) |
| **Jurisdiction** | |
| United States of America | 30 (45%) |
| United Kingdom | 23 (34%) |
| Europe | 6 (9.0%) |
| Australia and New Zealand | 4 (6.0%) |
| Asia | 3 (4.5%) |
| Canada | 2 (3.0%) |
| **Methodology** | |
| Review | 23 (34%) |
| Qualitative | 21 (32%) |
| Quantitative | 9 (14%) |
| Mixed | 7 (11%) |
| Methods | 8 (12%) |
| **Population** | |
| Long-term care | 51 (75%) |
| Geriatric acute care or mixed | 27 (25%) |

###### Supplementary Table 4. PRISMA Abstract Checklist for systematic review

| **Topic** | **No.** | **Item** | **Reported?** |
| --- | --- | --- | --- |
| **TITLE** |  |  |  |
| **Title** | 1 | Identify the report as a systematic review. | Yes |
| **BACKGROUND** |  |  |  |
| **Objectives** | 2 | Provide an explicit statement of the main objective(s) or question(s) the review addresses. | Yes |
| **METHODS** |  |  |  |
| **Eligibility criteria** | 3 | Specify the inclusion and exclusion criteria for the review. | Yes |
| **Information sources** | 4 | Specify the information sources (e.g. databases, registers) used to identify studies and the date when each was last searched. | Yes |
| **Risk of bias** | 5 | Specify the methods used to assess risk of bias in the included studies. | No |
| **Synthesis of results** | 6 | Specify the methods used to present and synthesize results. | Yes |
| **RESULTS** |  |  |  |
| **Included studies** | 7 | Give the total number of included studies and participants and summarise relevant characteristics of studies. | Yes |
| **Synthesis of results** | 8 | Present results for main outcomes, preferably indicating the number of included studies and participants for each. If meta-analysis was done, report the summary estimate and confidence/credible interval. If comparing groups, indicate the direction of the effect (i.e. which group is favoured). | Yes |
| **DISCUSSION** |  |  |  |
| **Limitations of evidence** | 9 | Provide a brief summary of the limitations of the evidence included in the review (e.g. study risk of bias, inconsistency and imprecision). | No |
| **Interpretation** | 10 | Provide a general interpretation of the results and important implications. | Yes |
| **OTHER** |  |  |  |
| **Funding** | 11 | Specify the primary source of funding for the review. | No |
| **Registration** | 12 | Provide the register name and registration number. | No |

*From:* Page MJ, McKenzie JE, Bossuyt PM, Boutron I, Hoffmann TC, Mulrow CD, et al. The PRISMA 2020 statement: an updated guideline for reporting systematic reviews. MetaArXiv. 2020, September 14. DOI: 10.31222/osf.io/v7gm2. For more information, visit: <www.prisma-statement.org>

######
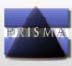
Supplementary Table 5. PRISMA 2020 Checklist for systematic review

| **Section and Topic** | **Item #** | **Checklist item** | **Location where item is reported** |
| --- | --- | --- | --- |
| **TITLE** | | |  |
| Title | 1 | Identify the report as a systematic review. | Title page |
| **ABSTRACT** | | |  |
| Abstract | 2 | See the PRISMA 2020 for Abstracts checklist. | Cf. completed PRISMA Abstract checklist |
| **INTRODUCTION** | | |  |
| Rationale | 3 | Describe the rationale for the review in the context of existing knowledge. | Background, paragraph 3 |
| Objectives | 4 | Provide an explicit statement of the objective(s) or question(s) the review addresses. | Background paragraph 4 |
| **METHODS** | | |  |
| Eligibility criteria | 5 | Specify the inclusion and exclusion criteria for the review and how studies were grouped for the syntheses. | Methods, paragraph 3 |
| Information sources | 6 | Specify all databases, registers, websites, organisations, reference lists and other sources searched or consulted to identify studies. Specify the date when each source was last searched or consulted. | Methods, paragraph 3; Supplementary methods |
| Search strategy | 7 | Present the full search strategies for all databases, registers and websites, including any filters and limits used. | Methods, paragraph 3; Supplementary methods |
| Selection process | 8 | Specify the methods used to decide whether a study met the inclusion criteria of the review, including how many reviewers screened each record and each report retrieved, whether they worked independently, and if applicable, details of automation tools used in the process. | Methods, paragraph 3 |
| Data collection process | 9 | Specify the methods used to collect data from reports, including how many reviewers collected data from each report, whether they worked independently, any processes for obtaining or confirming data from study investigators, and if applicable, details of automation tools used in the process. | Methods, paragraph 4 |
| Data items | 10a | List and define all outcomes for which data were sought. Specify whether all results that were compatible with each outcome domain in each study were sought (e.g. for all measures, time points, analyses), and if not, the methods used to decide which results to collect. | Methods, paragraph 4 |
|  | 10b | List and define all other variables for which data were sought (e.g. participant and intervention characteristics, funding sources). Describe any assumptions made about any missing or unclear information. | Methods, paragraph 4 |
| Study risk of bias assessment | 11 | Specify the methods used to assess risk of bias in the included studies, including details of the tool(s) used, how many reviewers assessed each study and whether they worked independently, and if applicable, details of automation tools used in the process. | N/A |
| Effect measures | 12 | Specify for each outcome the effect measure(s) (e.g. risk ratio, mean difference) used in the synthesis or presentation of results. | N/A |
| Synthesis methods | 13a | Describe the processes used to decide which studies were eligible for each synthesis (e.g. tabulating the study intervention characteristics and comparing against the planned groups for each synthesis (item #5)). | Methods, paragraph 4 |
|  | 13b | Describe any methods required to prepare the data for presentation or synthesis, such as handling of missing summary statistics, or data conversions. | Methods, paragraph 5 |
|  | 13c | Describe any methods used to tabulate or visually display results of individual studies and syntheses. | Methods, paragraph 5 |
|  | 13d | Describe any methods used to synthesize results and provide a rationale for the choice(s). If meta-analysis was performed, describe the model(s), method(s) to identify the presence and extent of statistical heterogeneity, and software package(s) used. | Methods, paragraph 5 |
|  | 13e | Describe any methods used to explore possible causes of heterogeneity among study results (e.g. subgroup analysis, meta-regression). | Methods, paragraph 5 |
|  | 13f | Describe any sensitivity analyses conducted to assess robustness of the synthesized results. | N/A |
| Reporting bias assessment | 14 | Describe any methods used to assess risk of bias due to missing results in a synthesis (arising from reporting biases). | N/A |
| Certainty assessment | 15 | Describe any methods used to assess certainty (or confidence) in the body of evidence for an outcome. | N/A |
| **RESULTS** | | |  |
| Study selection | 16a | Describe the results of the search and selection process, from the number of records identified in the search to the number of studies included in the review, ideally using a flow diagram. | Results, paragraph 2; Supplementary Figure 2 |
|  | 16b | Cite studies that might appear to meet the inclusion criteria, but which were excluded, and explain why they were excluded. | Supplementary Figure 2 |
| Study characteristics | 17 | Cite each included study and present its characteristics. | Supplementary Table 2 |
| Risk of bias in studies | 18 | Present assessments of risk of bias for each included study. | N/A |
| Results of individual studies | 19 | For all outcomes, present, for each study: (a) summary statistics for each group (where appropriate) and (b) an effect estimate and its precision (e.g. confidence/credible interval), ideally using structured tables or plots. | N/A |
| Results of syntheses | 20a | For each synthesis, briefly summarise the characteristics and risk of bias among contributing studies. | N/A |
|  | 20b | Present results of all statistical syntheses conducted. If meta-analysis was done, present for each the summary estimate and its precision (e.g. confidence/credible interval) and measures of statistical heterogeneity. If comparing groups, describe the direction of the effect. | N/A |
|  | 20c | Present results of all investigations of possible causes of heterogeneity among study results. | N/A |
|  | 20d | Present results of all sensitivity analyses conducted to assess the robustness of the synthesized results. | N/A |
| Reporting biases | 21 | Present assessments of risk of bias due to missing results (arising from reporting biases) for each synthesis assessed. | N/A |
| Certainty of evidence | 22 | Present assessments of certainty (or confidence) in the body of evidence for each outcome assessed. | N/A |
| **DISCUSSION** | | |  |
| Discussion | 23a | Provide a general interpretation of the results in the context of other evidence. | Discussion, paragraphs 2-3 |
|  | 23b | Discuss any limitations of the evidence included in the review. | N/A |
|  | 23c | Discuss any limitations of the review processes used. | Discussion, paragraph 7 |
|  | 23d | Discuss implications of the results for practice, policy, and future research. | Discussion, paragraphs 4-5 |
| **OTHER INFORMATION** | | |  |
| Registration and protocol | 24a | Provide registration information for the review, including register name and registration number, or state that the review was not registered. | Methods, paragraph 3 |
|  | 24b | Indicate where the review protocol can be accessed, or state that a protocol was not prepared. | N/A |
|  | 24c | Describe and explain any amendments to information provided at registration or in the protocol. | N/A |
| Support | 25 | Describe sources of financial or non-financial support for the review, and the role of the funders or sponsors in the review. | Declarations, section on Funding |
| Competing interests | 26 | Declare any competing interests of review authors. | Declarations, section on Competing interests |
| Availability of data, code and other materials | 27 | Report which of the following are publicly available and where they can be found: template data collection forms; data extracted from included studies; data used for all analyses; analytic code; any other materials used in the review. | Declarations, section on Availability of data and materials |

*From: Page MJ, McKenzie JE, Bossuyt PM, Boutron I, Hoffmann TC, Mulrow CD, et al. The PRISMA 2020 statement: an updated guideline for reporting systematic reviews. BMJ 2021;372:n71. doi: 10.1136/bmj.n71*

*For more information, visit:* [www.prisma-statement.org](file:///C:\Users\p0120505\Downloads\www.prisma-statement.org)

## Supplementary Figures

###### Supplementary Figure 1. Initial conceptual framework and interaction map

**
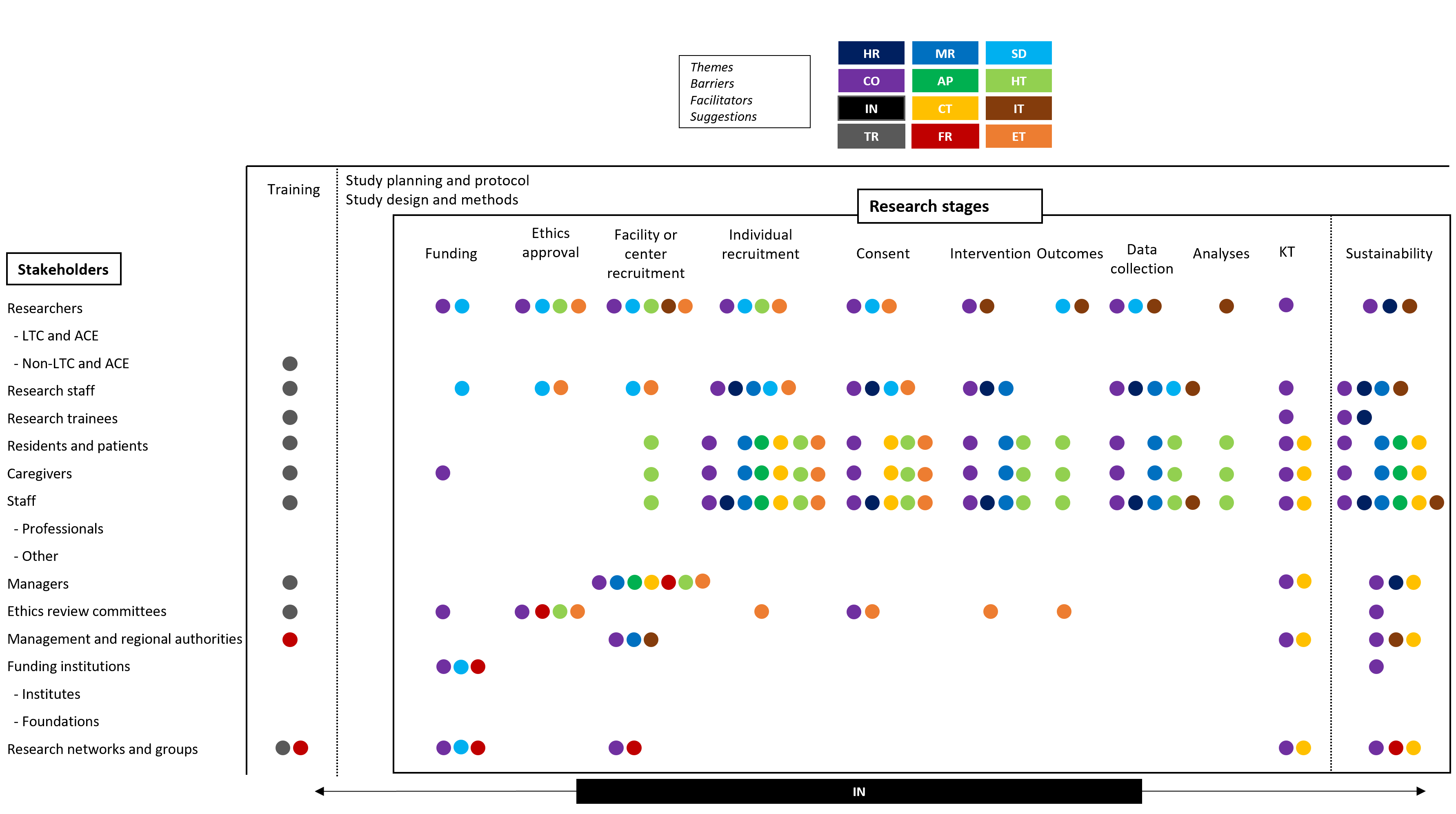
**

###### Supplementary Figure 2. PRISMA flow chart for the selection of articles

**
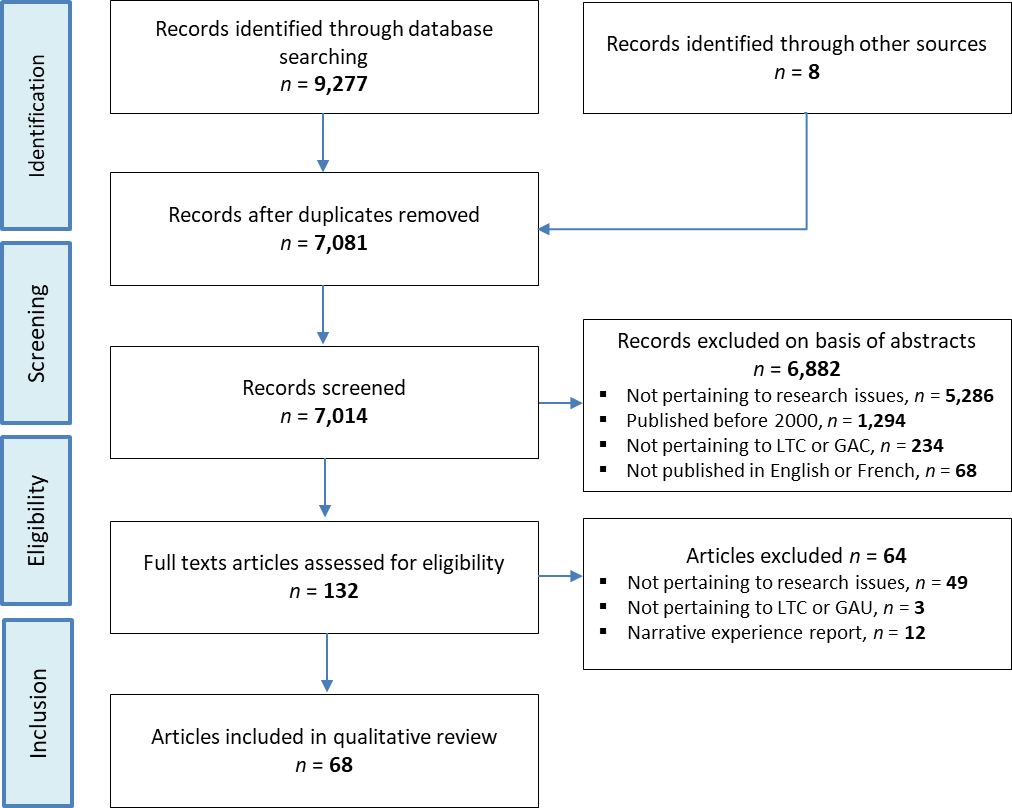
**

###### Supplementary Figure 3. Perception of stakeholders, research stages, and themes as facilitator or barriers


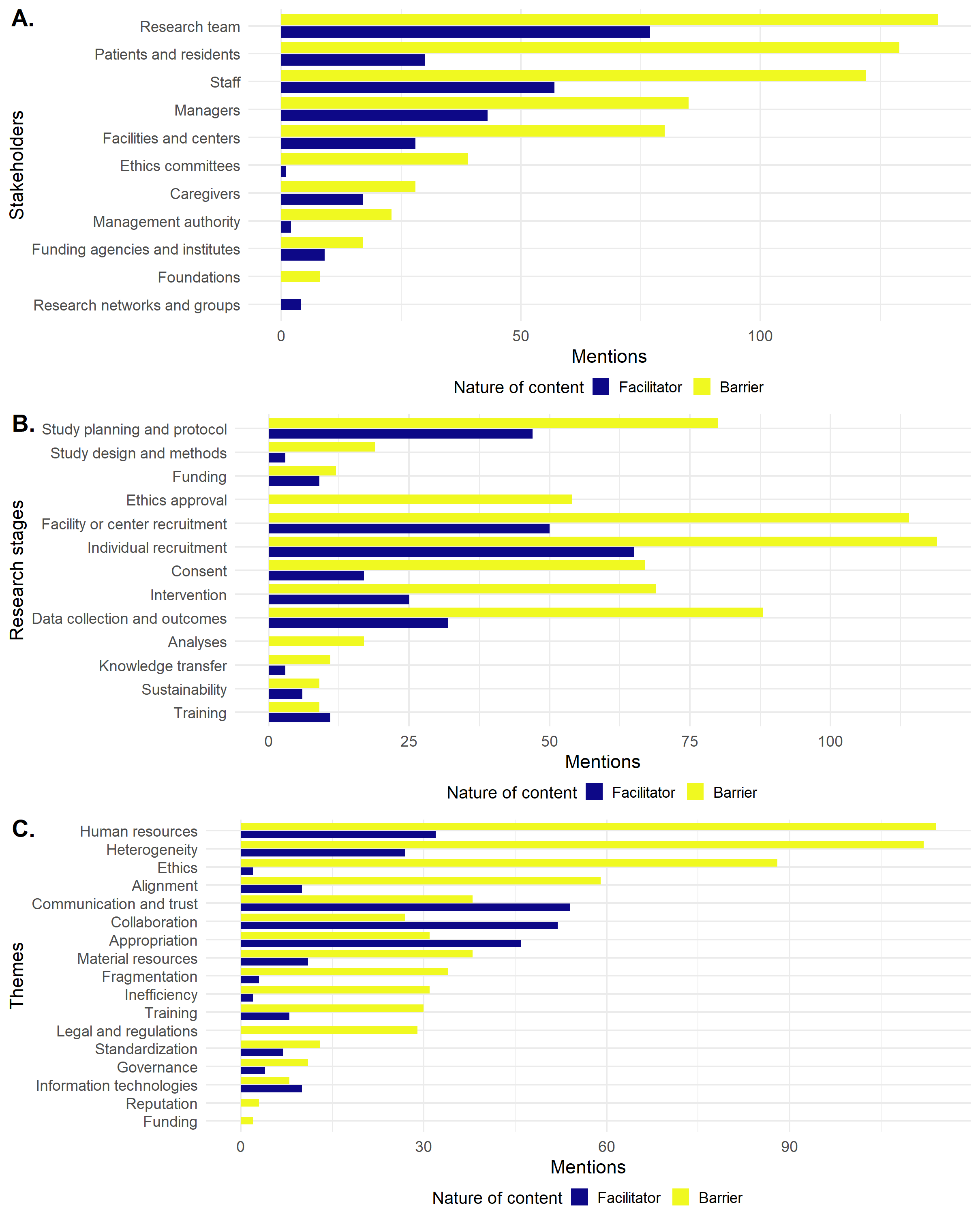


**Legend.** There were 668 instances of barriers and 268 of facilitators related to stakeholders, research stages, and themes. **A.** Stakeholders, **B.** Research stages, **C.** Themes.
